# Enantiomers of 2-methylglutamate and 2-methylglutamine selectively impact mouse brain metabolism and behavior

**DOI:** 10.1038/s41598-021-87569-1

**Published:** 2021-04-14

**Authors:** Adam M. Wawro, Chandresh R. Gajera, Steven A. Baker, Robert K. Leśniak, Curt R. Fischer, Nay L. Saw, Mehrdad Shamloo, Thomas J. Montine

**Affiliations:** 1 Department of Pathology, Stanford University, Stanford, USA; 2 ChEM-H, Stanford University, Stanford, USA; 3 Behavioral and Functional Neuroscience Laboratory, Stanford University, Stanford, USA; 4 Department of Neurosurgery, Stanford University, Stanford, USA

**Keywords:** Neurochemistry, Pharmacology, Mass spectrometry

## Abstract

Imbalance of excitatory and inhibitory neurotransmission is implicated in a wide range of psychiatric and neurologic disorders. Here we tested the hypothesis that insertion of a methyl group on the stereogenic alpha carbon of l-Glu or l-Gln would impact the γ-aminobutyric acid (GABA) shunt and the glutamate-glutamine cycle. (*S*)-2-methylglutamate, or (*S*)-2MeGlu, was efficiently transported into brain and synaptosomes where it was released by membrane depolarization in a manner equivalent to endogenous l-Glu. (*R*)-2MeGlu was transported less efficiently into brain and synaptosomes but was not released by membrane depolarization. Each enantiomer of 2MeGlu had limited activity across a panel of over 30 glutamate and GABA receptors. While neither enantiomer of 2MeGlu was metabolized along the GABA shunt, (*S*)-2MeGlu was selectively converted to (*S*)-2-methylglutamine, or (*S*)-2MeGln, which was subsequently slowly hydrolyzed back to (*S*)-2MeGlu in brain. rac-2MeGln was also transported into brain, with similar efficiency as (*S*)-2MeGlu. A battery of behavioral tests in young adult wild type mice showed safety with up to single 900 mg/kg dose of (*R*)-2MeGlu, (*S*)-2MeGlu, or rac-2MeGln, suppressed locomotor activity with single ≥ 100 mg/kg dose of (*R*)-2MeGlu or (*S*)-2MeGlu. No effect on anxiety or hippocampus-dependent learning was evident. Enantiomers of 2MeGlu and 2MeGln show promise as potential pharmacologic agents and imaging probes for cells that produce or transport l-Gln.

## Introduction

**Figure 1:**
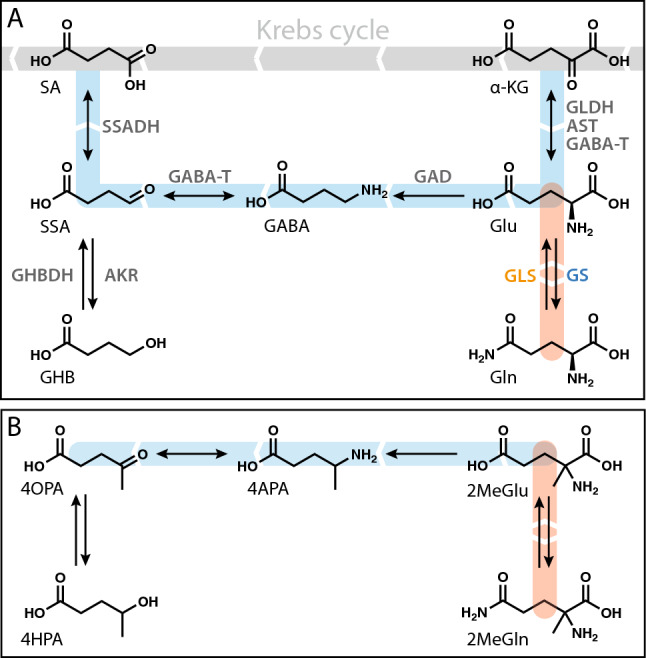
l-Glutamate is involved in the GABA shunt (blue arrows) off of the Krebs cycle and in the glutamate-glutamine cycle (red arrows) (**A**). Glutamate amidation proceeds primarily in astrocytes (blue font), while the reverse reaction occurs in neurons (orange font). For simplicity the transport steps are not shown. Hypothetical metabolic scheme of 2MeGlu into methyl analogues of Glu metabolites (**B**). SA and α-KG have no exact analogues and are not shown in the figure. *AKR* aldo–keto reductase, *AST* aspartate transaminase, *GABA-T* GABA transaminase, *GAD* glutamate decarboxylase, *GHBDH* gamma-hydroxybutyrate dehydrogenase, *GLDH* glutamate dehydrogenase, *GLS* glutaminase, *GS* glutamine synthetase, *SSADH* succinic semialdehyde dehydrogenase, *GABA* γ-aminobutyric acid, *GHB* γ-hydroxybutyric acid, *Gln* l-glutamine, *Glu* l-glutamate, *α-KG* α-ketoglutarate, *SA* succinic acid, *SSA* succinic semialdehyde.

The amino acid l-glutamine and the neurotransmitters l-glutamate and γ-aminobutyric acid (GABA) are linked metabolically via the glutamate-glutamine cycle and the GABA shunt (Fig. 1)^1,2^. Imbalance of the complex metabolic interplay between excitatory (mostly glutamatergic) and inhibitory (mostly GABAergic) neurotransmission has been implicated in a wide range of psychiatric and neurologic disorders. Inherited deficiencies in the glutamate-glutamine cycle, either glutamine synthetase (GS) deficiency (OMIM 610015) or glutaminase deficiency (OMIM 138280), yield neonatal-infantile epileptic encephalopathy and early death^3,4^. Inherited deficiencies within the GABA shunt include SSADH deficiency (OMIM 271980) and GABA-transaminase deficiency (OMIM 613163) that are characterized by language and motor delay, cognitive impairment, ataxia, autistic behaviors, hallucinations, and epilepsy in both children and adults^5,6^, with elevated γ-hydroxybutyrate (GHB) and/or GABA^7,8^. Prevalent diseases are proposed to derive at least in part from more subtle imbalance of excitatory and inhibitory neurotransmission, including mood disorders, some forms of psychosis, epilepsy, Alzheimer’s disease, and Parkinson’s disease^9^. The prospect of restoring balance to these opposing systems has been the focus of many candidate therapeutics, some of which have gone on to wide-spread clinical application: receptor specific agonists, antagonists, and allosteric modulators, transporter inhibitors, gene transfer, and most recently cell therapy^10,11^. None of these approaches target the metabolic balance of the GABA shunt or the glutamate-glutamine cycle, and as yet none has had a major impact on alleviating the common psychiatric or neurodegenerative diseases listed above. Here we tested the hypothesis that insertion of a methyl group on the stereogenic alpha carbon of l-glutamate or l-glutamine would provide novel tools to modulate the metabolic balance within the the GABA shunt or the glutamate-glutamine cycle, and perhaps have utility as molecular imaging agents or drugs that influence behavior.

In addition to 2MeGlu, we evaluated its hypothetical metabolites, i.e., analogues of the major Glu metabolites methylated at the carbon atom located at the α position in Glu; these include: 2-methylglutamine (2MeGln, the Gln analogue), 4-aminopentanoic acid (4APA, the GABA analogue), 4OPA (4-oxopentanoic acid or levulinic acid, the SSA analogue), and 4-hydroxypentanoic acid (4HPA, the GHB analogue). α-KG and SA have no corresponding methylated analogues; the presence of the hypothetical carbon atom at the α position would not be possible without breaking the existing C–C bonds. Since α-KG and SA connect Glu to the Krebs cycle, 2MeGlu cannot enter the cycle in a Glu-like manner.

## Results

We used LC–MS/MS with HILIC and chiral HILIC-like chromatography to resolve all compounds of interest, including their structural isomers, present in biological matrices over a wide range of concentrations^12^. Introduction of a methyl group on the α carbon drastically reduced the ability of the stationary phase to resolve enantiomers compared to natural amino acids, nevertheless we achieved baseline resolution of 2MeGlu and 4APA enantiomers without prior derivatization (Supplementary Figure S1).

**Figure 2:**
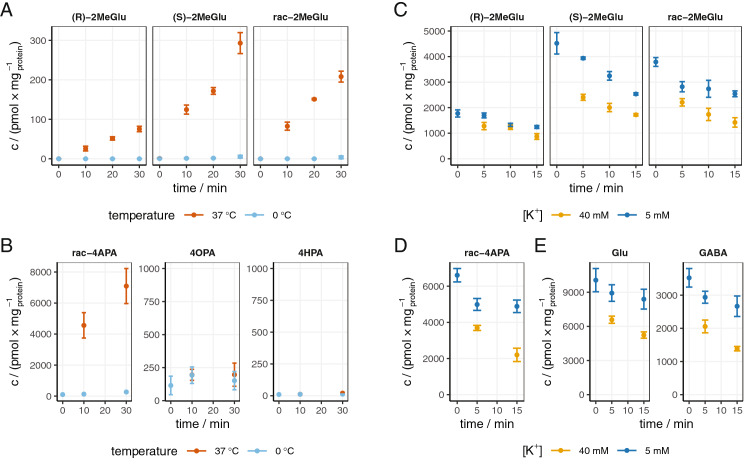
Synaptosomal accumulation of 2MeGlu (**A**) and 2Me analogues of the GABA shunt metabolic pathway (**B**). Synaptosomes were incubated with 10 μM substrates at 0 °C or 37 °C and the uptake was monitored over time. Error bars represent s.e.m., n = 3. Potassium-induced release of Synaptosomal 2MeGlu (**C**), 4APA (**D**) and endogenous Glu and GABA (**E**). Synaptosomes were preincubated with 100 μM substrate (**C**,**D** only) for 15 min at 37 °C. The pellet was washed, resuspended in normal or high-potassium KRP buffer and incubated for the times indicated at 37 °C. Data are shown as mean ± SEM, n = 3.

Evaluation of the neurotransmitter-like properties was performed using mouse cerebral synaptosomes, a preparation of functional, mostly presynaptic, nerve terminals. Uptake of both enantiomers of 2MeGlu was temperature-dependent (Fig. 2A), although the *S* enantiomer (analogous to l-2MeGlu) was approximately threefold faster than the *R* enantiomer (analogous to d-2MeGlu). Among the potential 2MeGlu metabolites, only rac-4APA was transported into synaptosomes, while 4OPA and 4HPA were not (Fig. 2B). We tested if 2MeGlu was in the neurotransmitter pool by subjecting preloaded synaptosomes to high potassium concentration-induced membrane depolarization. Both enantiomers of 2MeGlu were transported into synaptosomes during preloading; however, their potassium-induced release from vesicular storage was different (Fig. 2C). Synaptosomal concentration of the *R* enantiomer was not significantly altered by depolarization, whereas the *S* enantiomer was released upon depolarization in a manner very similar to endogenous Glu (Fig. 2E), as indicated by the relative difference of the substrate concentrations in the samples incubated under the low- and high-potassium conditions. When tested under identical conditions, rac-4APA exhibited high potassium concentration-dependent release (Fig. 2D) that was similar to endogenous GABA (Fig. 2E). Thus, (*R*)- and (*S*)-2MeGlu and rac-4APA were transported into synaptosomes, but only (*S*)-2MeGlu and at least one enantiomer of 4APA entered the neurotransmitter pool.

Initial metabolic assays also were performed in mouse cerebral synaptosomes, which are known to contain residual astrocytic components comprising < 5% of total terminals by electron microscopic or flow cytometric detection (Supplementary Figure S2)^13,14^. Synaptosomal concentration of rac-2MeGlu decreased slowly over time with an estimated half life of 21 min, and was not associated with the concurrent appearance of 4APA or any other 2Me analogue within the GABA shunt, indicating that the 2Me analogues were not efficiently metabolized by this pathway’s enzymes. We hypothesized that 2MeGlu might have been metabolized to 2MeGln and/or released back into the extrasynaptosomal space (Supplementary Figure S2). Indeed, rapid formation of 2MeGln along with a steady release of 2MeGlu were observed and accounted for the total concentration of all 2Me species over time, i.e., the total concentration of both 2MeGlu and 2MeGln did not change significantly over time (P = 0.70, repeated measures one-way ANOVA, n = 5) (Supplementary Figure S2), indicating that the only major metabolite of 2MeGlu in mouse synaptosomes was 2MeGln.

**Figure 3:**
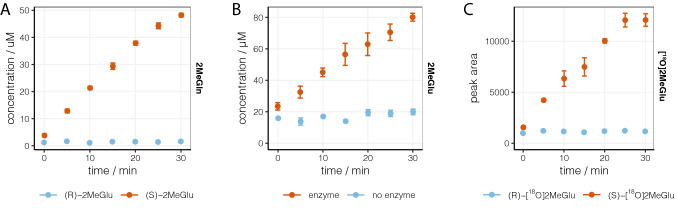
(**A**) Stereospecific conversion of 2MeGlu enantiomers by human glutamine synthetase. Reaction was performed with 10 mM substrate and 5 ng/mL enzyme in the presence of 10 mM ATP, 20 mM MgCl_2_, 40 mM NH_4_Cl, 25 mM 2-mercaptoethanol in 100 mM imidazole pH 7.0 buffer^18^. (**B**) Conversion of rac-2MeGln by human glutaminase GLS1. Reaction was performed with 10 mM substrate and 5 ng/mL enzyme in phosphate pH 7.4 buffer^19^. (**C**) Relative concentrations of (*R*)- and (*S*)-[^18^O]2MeGlu generated in the GLS1 hydrolysis assay. Data are shown as mean ± SEM, n = 3.

GS is responsible for brain metabolism of Glu to Gln. Consistent with the synaptosome results, 2MeGlu was converted by human GS to 2MeGln in the presence of ammonium ions and ATP, but the reaction was highly stereoselective towards the *S* enantiomer (Fig. 3A). This is another stereochemical difference with Glu, both enantiomers of which are amidated by GS^15^. Our results are consistent with the previous work that showed the reaction of rac-2MeGlu with GS in the presence of ATP releases 50% of inorganic phosphate obtained under identical conditions with l-Glu^16^. The reverse reaction was tested in vitro using human glutaminase GLS1, a phosphate-dependent amidohydrolase, which accounts for over 50% of glutaminase activity in the brain^17^. In order to overcome the analytical interference caused by traces of 2MeGlu present in synthetic 2MeGln, catalytic hydrolysis was performed in H_2_^18^O-enriched buffer. Simultaneous detection of 2MeGlu and 2MeGlu-^18^O allowed us to distinguish between the newly formed 2MeGlu and any preexisting impurity, and thereby quantify exclusively the former (Fig. 3B). The appearance of 2MeGlu in the samples containing rac-2MeGln indicated that at least one enantiomer of 2MeGln is a substrate for human GLS1, albeit relatively slow; the apparent reaction rate was approximately 35-fold lower compared to that of Gln (65 μM/min vs 1.9 μM/min). Chiral analysis of the isotope-labeled hydrolysis products showed that the *S* enantiomer was the exclusive product of the GLS1-catalyzed reaction (Fig. 3C).

**Figure 4:**
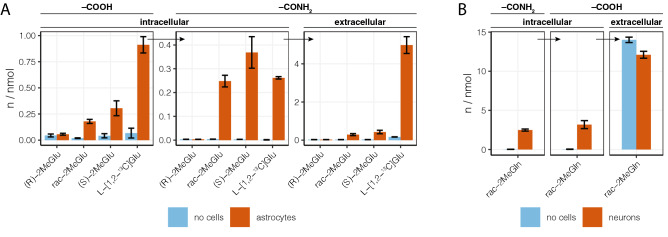
Cell metabolism of 2MeGlu and 2MeGln. Data are shown as mean ± SEM, n = 3. Horizontal arrows represent an uptake–conversion–release cycle. (**A**) Astrocyte uptake, amidation, and release of 2MeGlu and its amide derivative, 2MeGln. Total intra- and extracellular amounts of 2MeGlu or [1,2-^13^C]Glu (–COOH) and 2MeGln or [1,2-^13^C]Gln (–CONH_2_) were determined in cells and media. Primary astrocyte cultures were incubated in the presence of 1 mM of racemic or enantiopure 2MeGlu or l-Glu for 2 h at 37 °C along with cell-free controls. (**B**) Neuronal uptake, hydrolysis, and release of 2MeGln and its hydrolyzed counterpart 2MeGlu. Total intra- and extracellular amounts of 2MeGln (–CONH_2_) and 2MeGlu (–COOH) were determined in cells and media. Primary neuron cell cultures were incubated in the presence of 1 mM 2MeGln for 2 h at 37 °C along with cell-free controls.

Incubation of primary murine cerebral astrocytes with each enantiomer of 2MeGlu confirmed the stereoselective nature of 2MeGln formation observed in synaptosomes and with GS in vitro (Fig. 4). The *S* enantiomer was transported into astrocytes and then converted into 2MeGln. In contrast, the *R* enantiomer was not detected within astrocytes in significant amounts, and no conversion to 2MeGln was detected. Comparison with isotopically labeled l-[1,2-^13^C]Glu, the endogenous substrate, revealed that the intracellular concentrations of 2MeGln and [1,2-^13^C]Gln, made from (*S*)-2MeGlu and l-[1,2-^13^C]Glu respectively, were similar (0.35 nmol/well vs 0.26 nmol/well); however, the extracellular concentrations were an order of magnitude different (0.4 nmol/well vs 5.0 nmol/well), suggesting that 2MeGln was released from astrocytes at a much lower rate compared to [1,2-^13^C]Gln. Samples where racemic 2MeGlu was used as the substrate followed the pattern of the *S* enantiomer, although at concentrations consistently reduced 60–70%.

Enzymatic hydrolysis of 2MeGln back to 2MeGlu to complete the glutamate-glutamine cycle was tested in murine primary cerebral cortical neuron cultures (Fig. 4C). 2MeGlu was detected in the mouse neurons, although it was unclear whether it was a product of intracellular enzymatic hydrolysis of 2MeGln or direct transport from media, since traces of 2MeGlu were present in the 2MeGln synthetic product. The uptake of both enantiomers of 2MeGlu observed in synaptosomes and hydrolysis of at least one enantiomer of 2MeGln by human GLS1 suggested that both mechanisms contribute to the intracellular presence of 2MeGlu in neuron cultures. An identical experiment was performed in primary cerebral cortical neuron cultures with isotopically labeled l-[1,2-^13^C]Gln for comparison (data not shown). Intracellular [1,2-^13^C]Gln was twofold lower than 2MeGln in corresponding samples, while [1,2-^13^C]Glu was over fivefold lower than 2MeGlu in cells. Together with the presence of extracellular [1,2-^13^C]Glu, these results suggest that [1,2-^13^C]Gln is enzymatically hydrolyzed and transported out of the cell at a higher rate than 2MeGln.

In summary, the above in vitro, synaptosome, and primary cell culture experiments show that 2MeGlu and 2MeGln are transported in and out of neurons and astrocytes, similar to their non-methylated endogenous analogues, while having more restricted, stereochemically-dependent, metabolic pathways. (*S*)-2MeGlu and (*S*)-2MeGln were shown to be substrates of GS and GLS1, respectively, while their *R* enantiomers were not. In addition, the relatively low rate of extracellular release of endogenously synthesized 2MeGln suggests partial trapping of the amidation product in primary astrocytes where it was generated.

Since GS is important to proliferation of some cancer cells^20^, we determined the impact of both 2MeGlu enantiomers and rac-2MeGln on proliferation of glutamine-dependent MDA-MB-231 and SK-OV-3, and glutamine-independent MCF-7^21,22^. Cell viability assays were conducted by BPS Bioscience and showed that none of the test compounds effectively inhibited cell proliferation (IC_50_ > 100 μM) in any of the three cell lines (Supplementary Figure S3). These results aligned with our GLS1 inhibition assay, where none of the compounds displayed significant inhibitory activity (IC_50_ > 1 mM) (Supplementary Figure S3).

**Figure 5:**
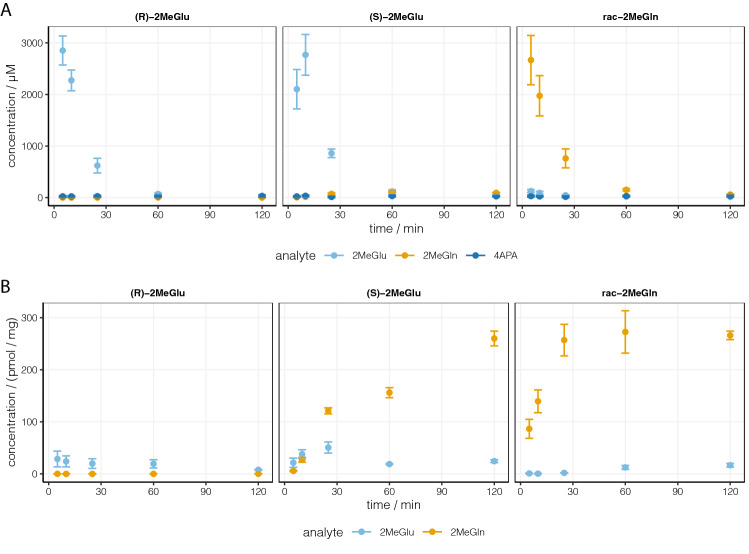
Pharmacokinetics of 2Me compounds and their metabolites measured in mouse serum (**A**) and brain (**B**) after IP injection of 100 mg/kg of test compounds (in columns). Concentrations are expressed per volume in the serum samples and per protein content in the brain samples. Data are shown as mean ± SEM, n = 3.

We next examined pharmacokinetics of 2Me compounds in mouse blood and brain (Fig. 5). All compounds reached their maximum serum concentration within 15 min of intraperitoneal (IP) administration and were practically cleared from blood within 60 min (Fig. 5A). All tested compounds entered the brain where their kinetic profiles differed substantially (Fig. 5B). (*R*)-2MeGlu brain concentration peaked within 5 min at 30 pmol/mg protein and decreased over time with an estimated half life of approximately 70 min. (*S*)-2MeGlu reached its maximum brain concentration of 50 pmol/mg protein after 25 min, while being actively converted into its only known metabolite, 2MeGln, which concentration rose monotonically to over 250 pmol/mg protein at 120 min. Thus, (*S*)-2MeGlu and its metabolite (*S*)-2MeGln accumulated in the brain as much as tenfold more than (*R*)-2MeGlu. Administered rac-2MeGln yielded peak brain concentration of 2MeGln more rapidly (25 min) than injection with (*S*)-2MeGlu, and then was relatively constant to 120 min. Interestingly, the concentrations of 2MeGln in mouse brain at the last time point (120 min) were almost identical whether administered (*S*)-2MeGlu or rac-2MeGln, indicating that (*S*)-2MeGlu is efficiently metabolized to 2MeGln in mouse brain and could act as a prodrug/precursor. A common feature observed for both (*S*)-2MeGlu and rac-2MeGln is the appearance of 2MeGlu at the 60 min and 120 min time points. Previously presented results from GLS1 enzymatic assay suggested that (*S*)-2MeGln undergoes slow enzymatic hydrolysis to (*S*)-2MeGlu. The pharmacokinetic experiment in mice aligns with that observation; the delayed appearance of 2MeGlu in the brain after the administered compounds have been cleared from blood indicates that 2MeGlu is slowly metabolized from the 2MeGln accumulated in brain, whether the original source was rac-2MeGln or (*S*)-2MeGlu. Finally, brain levels of endogenous Glu, Gln, or GABA also were determined as part of these experiments; two-way ANOVA showed that endogenous brain levels of Glu, GABA, or Gln were not significantly altered by either any of the three compounds administered at 100 mg/kg or the time after compound administration from 5 to 120 min (not shown).

We then explored the pharmacodynamics of enantiopure glutamate-mimicking compounds against a broad panel of glutamate and GABA receptors in both binding and functional assays (summarized in Table 1 and detailed in Supplementary Tables S1 to S5). We deliberately assayed 2MeGlu enantiomers for activity against both glutamate and GABA receptors in case of receptor class switching as occurs with α-methyldopamine^23,24^. Both compounds had weak to no significant activity against the large number of glutamate and GABA receptors screened. Interestingly, (*S*)-2MeGlu did not interact significantly with any of the tested receptors. (*R*)-2MeGlu exhibited weak antagonist behavior against GluN2A, one of four isoforms of the glutamate-binding subunit of *N*-methyl-d-aspartate (NMDA) receptors.

**Table 1:** Summary of glutamate and GABA receptor interaction assays. ‘↑’ or ‘↓’ arrows indicate agonist or antagonist activity, respectively. The number of arrows indicates IC50/EC50 in the range of 10–100 μM (one arrow), 1–10 μM (two arrows), or < 1 μM (three arrows). Interactions with EC_50_ or IC_50_ above 100 μM are marked as “–”.

Receptor name	Receptor type	Assay type	(*R*)-2MeGlu	(*S*)-2MeGlu
GluN2A	Ionotropic	Functional	↓	–
GluN2B	Ionotropic	Functional	–	–
GluN2C	Ionotropic	Functional	–	–
GluN2D	Ionotropic	Functional	–	–
GluA2	Ionotropic	Functional	–	–
mGluR1	Metabotropic	Binding	–	–
mGluR2	Metabotropic	Binding	–	–
mGluR5	Metabotropic	Binding	–	–
NMDA nonspecific	Ionotropic	Binding	–	–
AMPA nonspecific	Ionotropic	Binding	–	–
kainate nonspecific	Ionotropic	Binding	–	–
GABA_A_ α1β1γ2	Ionotropic	Functional	–	–
GABA_A_ α1β2γ2	Ionotropic	Functional	–	–
GABA_A_ α1β3γ2	Ionotropic	Functional	–	–
GABA_A_ α2β1γ2	Ionotropic	Functional	–	–
GABA_A_ α2β2γ2	Ionotropic	Functional	–	–
GABA_A_ α2β3γ2	Ionotropic	Functional	–	–
GABA_A_ α3β1γ2	Ionotropic	Functional	–	–
GABA_A_ α3β2γ2	Ionotropic	Functional	–	–
GABA_A_ α3β3γ2	Ionotropic	Functional	–	–
GABA_A_ α4β1γ2	Ionotropic	Functional	–	–
GABA_A_ α4β2γ2	Ionotropic	Functional	–	–
GABA_A_ α4β3γ2	Ionotropic	Functional	–	–
GABA_A_ α4β3δ	Ionotropic	Functional	–	–
GABA_A_ α5β1γ2	Ionotropic	Functional	–	–
GABA_A_ α5β2γ2	Ionotropic	Functional	–	–
GABA_A_ α5β3γ2	Ionotropic	Functional	–	–
GABA_A_ α6β1γ2	Ionotropic	Functional	–	–
GABA_A_ α6β2γ2	Ionotropic	Functional	–	–
GABA_A_ α6β3γ2	Ionotropic	Functional	–	–
GABA_B_ (B1/B2)	Metabotropic	Binding	–	–
GABA_B_ (B1a/B2)	Metabotropic	Binding	–	–
GABA_B_ (B1b/B2)	Metabotropic	Binding	–	–
GABA_B_ (B1/B2)	Metabotropic	Functional	–	–

**Figure 6:**
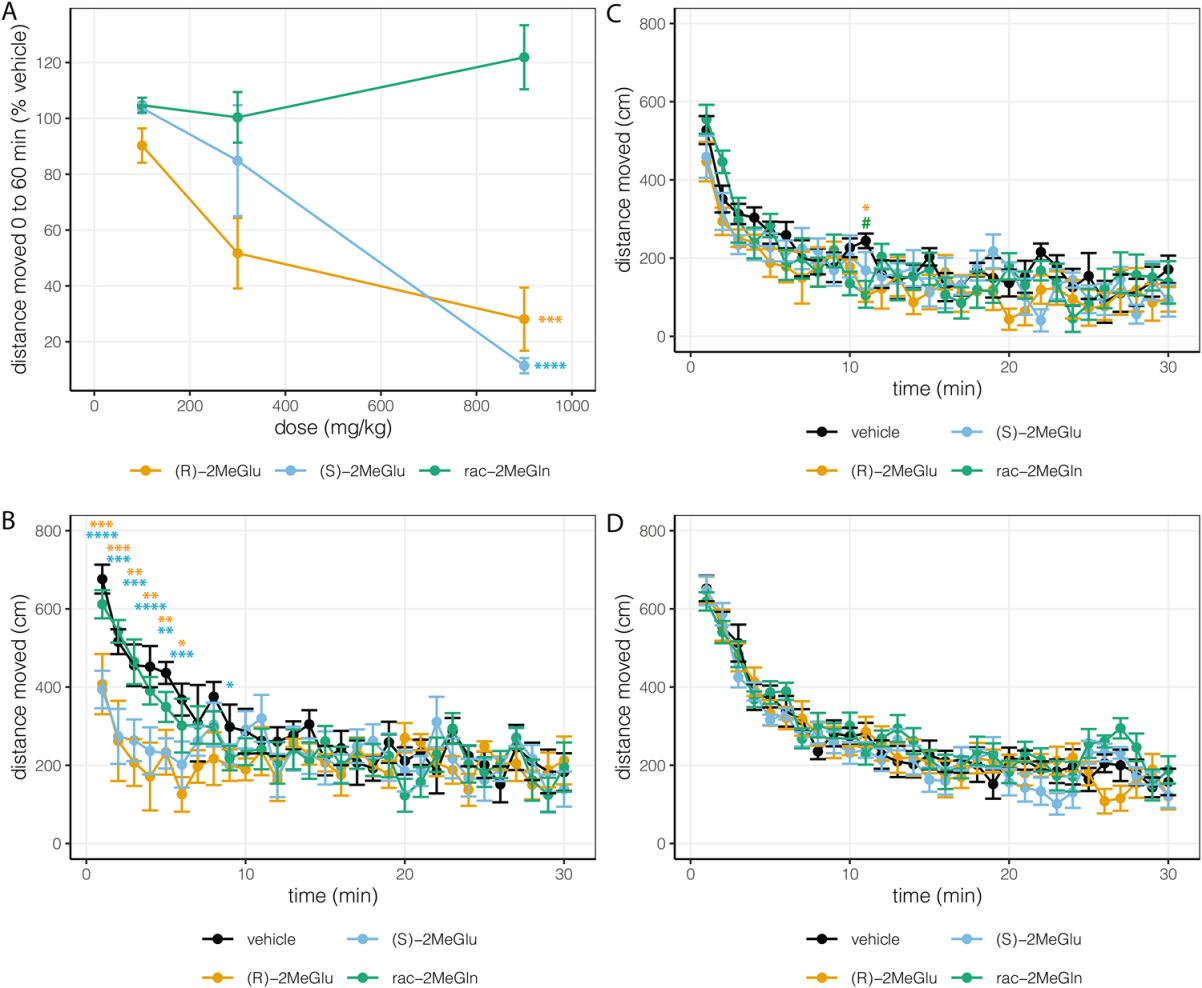
Exploratory and locomotor behavior in 2 month-old male C57Bl/6 mice was assessed by total distance moved in the novel cage test (**A**) or in the activity chamber (**B**–**D**). Mice were either injected and then immediately tested (acute groups in **A**–**C**), or injected daily for one week prior to testing (chronic groups in **D**). Data are shown as mean ± SEM. (**A**) Dose–response for each of the three compounds vs. total distance moved from 0 to 60 min in the novel cage test expressed as % of vehicle exposed mice (n = 3 mice per group). Two-way ANOVA had F_Interaction_ (4, 18) = 9.068, P = 0.0003; F_Dose_ (2, 18) = 14.91, P = 0.0002; and F_Compound_ (2, 18) = 21.89, P < 0.0001. Tukey’s multiple comparison test was significantly different for rac-2MeGln vs. (*R*)-2MeGlu (****P < 0.0001) and vs. (*S*)-2MeGlu (***P = 0.0002). (**B**) Time course of activity chamber distance moved following acute 100 mg/kg had two-way repeated measures ANOVA with F_Interaction_ (87, 580) = 1.879, P < 0.0001; F_Time_ (29, 580) = 11.44, P < 0.0001; and F_Exposure_ (3, 20) = 2.906, P = 0.0600 (n = 6 mice per group). Dunnett's multiple comparisons test had multiple early time points that were significantly different between vehicle and (*R*)-2MeGlu or between vehicle and (*S*)-2MeGlu (*P < 0.05, **P < 0.01, ***P < 0.001, ****P < 0.0001); vehicle was not significantly different from rac-2MeGln at any time point. (**C**) Time course of activity chamber distance moved following acute 30 mg/kg had two-way repeated measures ANOVA with F_Interaction_ (87, 580) = 0.9328, P = 0.6493; F_Time_ (29, 580) = 20.13, P < 0.0001; and F_Exposure_ (3, 20) = 1.805, P = 0.1786 (n = 6 mice per group). Dunnett's multiple comparisons test had one time point that was significantly different between vehicle and (*R*)-2MeGlu (*P < 0.05) or between vehicle and rac-2MeGln (^#^P < 0.05); vehicle was not significantly different from (*S*)-2MeGlu at any time point. (**D**) Time course of activity chamber distance moved following chronic 10 mg/kg per day for one week had two-way repeated measures ANOVA with F_Interaction_ (87, 986) = 1.22, P = 0.0912; F_Time_ (29, 986) = 62.35, P < 0.0001; and F_Exposure_ (3, 34) = 1.122, P = 0.3536; (n = 6 mice per group). One vehicle group mouse and one (*S*)-2MeGlu group mouse were outliers using the ROUT method; they were removed from analysis.

Our data suggested that 2MeGlu was a false neurotransmitter that also interfered with the glutamate-glutamine cycle. Investigators at the Stanford Behavioral and Functional Neuroscience Laboratory (SBFNL), who were blinded to the identity of the compounds being tested, performed a broad-based screen of safety and tolerability followed by tests of learning and memory, similar to the behavioral screen of transgenic mice with glutaminase deficiency^17^. Male 2 month-old healthy mice were injected with a single dose (100, 300, or 900 mg/kg IP) of (*R*)-2MeGlu, (*S*)-2MeGlu, or rac-2MeGln and then immediately assessed by novel cage observation and for survival. Only one mouse died 10 days after 900 mg/kg of (*R*)-2MeGlu. Distance moved in the novel cage was significantly decreased for both (*R*)-2MeGlu and (*S*)-2MeGlu (P < 0.001 for each, n = 3) in a dose- and time-dependent manner, indicating marked reduction in response to novelty/locomotor activity while remaining alert and responsive (Fig. 6A and Supplementary Figure S4). Next, we performed a behavioral test battery called SHIRPA (measures 23 sensorimotor functions and well being), activity chamber open field assessment, and then withdrawal from hot plate using new groups of male 2 month-old mice following a single injection of 100 or 30 mg/kg IP of one of the three compounds or vehicle (n = 6 per group). At the 100 mg/kg IP dose, there was no mortality, difference in latency to withdraw from hotplate, or difference on any SHIRPA score except locomotor activity for (*R*)-2MeGlu or (*S*)-2MeGlu (not shown). Distance moved in the activity chamber was significantly reduced for (*R*)-2MeGlu or (*S*)-2MeGlu compared to vehicle at all time points up to 6 min post injection with no significant difference in distance moved beyond 9 min; rac-2MeGln was not significantly different from vehicle at any time point (Fig. 6B). We repeated the acute exposure experiment in a separate set of mice now using 30 mg/kg or vehicle IP and observed modest but significant reduction in distance moved only at 11 min post injection with (*R*)-2MeGlu and rac-2MeGln (Fig. 6C); no significant differences on SHIRPA scores, or latency to withdraw from hot plate, were observed in the 30 mg/kg IP dose groups (not shown).

**Figure 7:**
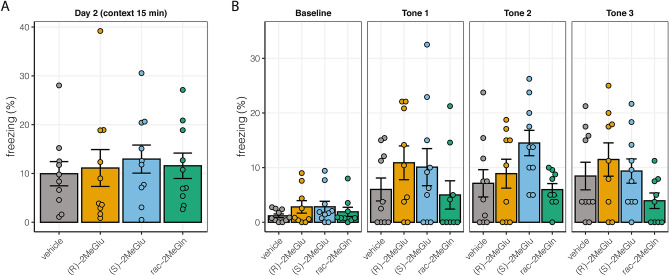
Behavioral effects of chronic 10 mg/kg/day IP dosing in 2 month-old male C57Bl/6 mice in fear conditioning tests on the fourth week of chronic exposure. (**A**) Freezing by the treatment group on Day 2 during contextual memory testing over 15 min. Data was not normally distributed. Kruskal–Wallis test had P approximately 0.7819. (**B**) Freezing at baseline and during each tone presentation on Day 3. Two-way repeated measures ANOVA had F_Interaction_ (9, 102) = 1.313, P = 0.24; F_Tone_ (3, 102) = 13.95, P < 0.0001; and F_Exposure_ (3, 34) = 2.015, P = 0.13. All bars represent mean ± SEM, n = 10 mice per group.

Subsequently, still-blinded experiments at SBFNL pursued behavioral impact of chronic low dose (*S*)-2MeGlu, (*R*)-2MeGlu, or rac-2MeGln in drug naïve, male, 2 month-old C57Bl/6 mice dosed with 10 mg/kg/day or vehicle IP for a month. Behavioral tests (n = 10 mice per group) were performed starting one week after chronic dosing and followed well established protocols: activity chamber and elevated plus maze (week 1 of testing), Y maze (week 2 of testing), Morris water maze (weeks 2 and 3 of testing), and fear conditioning (week 4 of testing)^25–30^. There was no mortality and no significant effect of treatment on body weight over the month, distance moved in the activity chamber (Fig. 6D), time spent in the open arms in the elevated plus maze (Supplementary Figure S5), spontaneous alternation in the Y maze (Supplementary Figure S6), or learning in the Morris water maze (Supplementary Figure S7). In the fear conditioning paradigm, all treatment groups showed equivalent learning of the tone-shock association on Day 1 (Supplementary Figure S8). There was no significant effect of treatments compared to the vehicle treated group in contextual recall on Day 2 (Fig. 7A), indicating comparable hippocampal memory recall among experimental groups, consistent with the Morris water maze results. Finally, there were no significant differences detected in freezing during the cued recall or tone presentation on Day 3 between drugs and vehicle treated groups (Fig. 7B, Supplementary Figure S9).

## Discussion

Methylation of the alpha carbon of neurotransmitter precursors has been used with l-dihydroxyphenylalanine (l-DOPA) to create Aldomet or α-methyldopa, one of the first and still used antihypertensives^31^, and with l-tryptophan to create a serotonergic PET imaging agent^32,33^. Racemic 2MeGlu is generally synthesized from levulinic acid^34–36^ and has been used in enzymatic studies^37–41^; as a potential effector of glutamate receptors^42–46^, transporters^47–51^, and brain tumor cell growth^52^; in tissue slices as a potential effector of dopamine release^53^, ammonia balance^54^, calcium uptake^55^, and metabotropic receptors^56^; and in animals to moderate induced circling behavior^57^. Enantiomers of 2MeGlu have been obtained by separation of racemate by a variety of methods^16,58–60^, and (*S*)-2MeGlu^61–63^ or (*R*)-2MeGlu^64^ have been synthesized by a variety of approaches. (*S*)-2MeGlu has been tested as an inhibitor of EAAT1/2 transporters^65,66^. As far as we are aware, despite the use of rac-2MeGlu as a congener in these experiments, mostly published from the 1950s to 1980s, no one has investigated the metabolic impact of enantiomers of 2MeGlu or 2MeGln in brain, or explored their potential enantiomer-specific activity in animals. Here, we tested the hypothesis that enantiomers of 2MeGlu or 2MeGln selectively modulate metabolism in the GABA shunt or glutamate-glutamine cycle, and may act as potentially useful pharmacologic or imaging agents.

By design, 2-MeGlu was not a substrate for GAD or GLDH and therefore progressed neither through the GABA shunt nor underwent oxidative deamination. The *S* enantiomer (L isomer by the convention used for natural amino acids) was avidly transported into synaptosomes and stored and released like l-Glu. While substituting for l-Glu in the neurotransmitter pool, (*S*)-2MeGlu did not have significant activity across a broad spectrum of glutamate and GABA receptors. In aggregate, these data support classifying (*S*)-2MeGlu as a false neurotransmitter. We demonstrated that only (*S*)-2MeGlu is a substrate for GS and thereby can enter the glutamate-glutamine cycle; however, (*S*)-2MeGlu was not an efficient substrate for the reverse reaction. The net effect in vivo was that the false neurotransmitter, (*S*)-2MeGlu, rapidly accumulated as (*S*)-2MeGln in brain, which was then slowly converted back to (*S*)-2MeGlu. Exposure to rac-2MeGln led to comparable but more rapid appearance of 2MeGln in brain, accompanied by slow generation of 2MeGlu over time. These properties lead us to propose that (*S*)-2MeGlu or 2MeGln might have useful applications as pharmacologic agents that alter the excitatory/inhibitory balance in brain, and perhaps as imaging agents for GS expressing or Gln transporting cells.

(*R*)-2MeGlu, as expected, also was not a substrate for GAD and did not progress in the GABA shunt. (*R*)-2MeGlu, although transported into synaptosomes, did not enter the neurotransmitter pool, suggesting that (*R*)-2MeGlu is a substrate for neuronal membrane transport but not synaptic vesicle transport. (*R*)-2MeGlu did have some weak activity at the GluN2A subunit of NMDA receptors. (*R*)-2MeGlu also was transported into mouse brain, but much less efficiently than the *S* enantiomer. (*R*)-2MeGlu was not a substrate for GS and so did not enter the glutamate-glutamine cycle. The net effect was that (*R*)-2MeGlu accesses the brain and synaptic, but not vesicular, compartments where it is isolated from the usual metabolism of l-Glu, l-Gln, or GABA, and has some weak activity at glutamate receptors.

The enantiomer-specific biochemical and metabolic properties of 2MeGlu or 2MeGln suggest that they may have useful applications as molecular imaging agents for GS expressing or Gln transporting cells, and perhaps as pharmacologic agents that could alter excitatory/inhibitory balance. We therefore pursued safety and behavioral testing in young adult, healthy, male mice. Acute, high doses of (*S*)-2MeGlu, (*R*)-2MeGlu, or rac-2MeGln had minimal mortality, and (*R*)-2MeGlu or (*S*)-2MeGlu caused temporary, marked reduction in response to novelty/locomotor activity while remaining alert and responsive to ambient noise and gentle touch. We speculate that this impressive, acute effect on mouse behavior observed at 100 mg/kg IP and higher doses may be due to temporary disturbance in glutamatergic neurotransmission from the inability of both enantiomers of 2MeGlu to be metabolized by GAD. The usefulness of such impaired response to novelty/locomotor activity is not clear but possibly could have utility in the management of patients undergoing procedures or imaging sessions. More work is needed to understand the impact of high dose on 2MeGlu enantiomers on neurotransmitter systems.

There are some limitations to our initial behavioral testing of enantiomers of 2MeGlu and 2MeGln. Although chronic, low dose exposure to compounds did not have a significant impact on behavioral tests, cumulative dosing varied over the month of exposure with our screening protocol. Future research is needed to determine more exact dose–response relationships in male mice and extend our experiments to include female mice, more detailed evaluation of impact on response to novelty vs. locomotor activity, and the effectiveness of these compounds in older mice and in models of neurological and psychiatric disorders proposed to derive in part from imbalance of excitatory and inhibitory neurotransmission.

## Methods

### Materials

(*R*)-2-methylglutamic acid, (*S*)-2-methylglutamic acid and rac-2-methylglutamine were made to order by Concept Life Sciences (Cheshire, UK) from racemate.

Buffer S (isotonic buffered sucrose solution): 0.32 M sucrose, 1 mM EDTA, 5 mM Tris–HCl pH 7.4. Buffer KRP (normal Krebs–Ringer phosphate buffer): 118.0 mM NaCl, 4.65 mM KCl, 1.23 mM CaCl_2_, 1.18 mM KH_2_PO_4_, 3.54 mM MgSO_4_, 15.64 mM Na_2_HPO_4_, 10 mM glucose. Buffer K-KRP (high-potassium Krebs–Ringer phosphate buffer): 84.3 mM NaCl, 40.5 mM KCl, 1.23 mM CaCl_2_, 1.18 mM KH_2_PO_4_, 3.54 mM MgSO_4_, 15.64 mM Na_2_HPO_4_, 10 mM glucose.

### Synaptosome preparation

Mouse cerebral synaptosomes were prepared according to published methods^14^. See Supplementary Methods for details. 500 μL aliquots of the synaptosome suspension were transferred to pre-chilled microcentrifuge tubes for experiments. Centrifugations were at 8000*g* for 4 min at 4 °C, unless stated otherwise, and all samples were stored at − 80 °C.

### Synaptosomal neurotransmitter uptake assay

To each tube, 5 μL of a test compound solution (final concentration 10 μM) in Buffer KRP or Buffer KRP alone were added. Samples were incubated at 4 °C or 37 °C for 1, 10, or 30 min and then immediately centrifuged and washed twice by resuspension–centrifugation with Buffer S (0.5 mL). Resulting pellets were resuspended in 170 μL of 0.1% aqueous formic acid and mixed with 330 μL of ACN.

### Potassium-induced neurotransmitter release assay

To each tube, 2.5 μL of a test compound solution (final concentration 100 μM) in Buffer KRP or Buffer KRP alone were added. Samples were incubated for 15 min at 37 °C, briefly chilled on ice, and then immediately centrifuged and washed twice with Buffer KRP. The resulting pellets were then resuspended in either Buffer KRP (500 μL) or Buffer K-KRP (500 μL). Samples were immediately centrifuged or incubated for 5, 10 or 15 min at 37 °C, then centrifuged and washed with Buffer KRP. Resulting pellets were resuspended in 400 μL of 60% ACN containing 2.5 μM Glu-d5 and 2.5 μM Gln-d5 as internal standards.

### Metabolism in the GABA shunt pathway in synaptosomes

To each tube, a 5 μL aliquot of a test compound solution (final concentration 100 μM) in Buffer KRP or Buffer KRP alone were added. The samples were incubated for 15 min at 37 °C, briefly chilled on ice, and then immediately centrifuged and washed twice by resuspension–centrifugation with Buffer S (0.5 mL).

The pellets were then resuspended in Buffer KRP (500 μL). Samples were then immediately centrifuged or incubated for 5, 10, 15, 20, 25 or 30 min at 37 °C and then centrifuged and washed by resuspension–centrifugation with Buffer S (0.5 mL). Resulting pellets were resuspended in 170 μL of 0.1% aqueous formic acid, mixed with 330 μL of ACN.

### Metabolism in the glutamate-glutamine cycle in synaptosomes

To each tube, 5 μL of 2MeGlu solution (final concentration 100 μM) in Buffer KRP or Buffer KRP alone were added. The samples were incubated for 15 min at 37 °C, briefly chilled on ice, and then immediately centrifuged and washed twice by resuspension–centrifugation with Buffer S (0.5 mL). The pellets were then resuspended in Buffer KRP (200 μL). Samples were then kept on ice or incubated for 10, 20, 30, 40 or 50 min at 37 °C and then chilled on ice. Then all samples were centrifuged, 200 μL of supernatants were collected, and the pellets were suspended in 200 μL of 80% ACN. Supernatants and pellet suspensions were diluted with 250 μL of ACN and 50 μL of 10 μM d5-Glu in water as internal standard.

### Catalysis of 2MeGlu C5-amidation by human glutamine synthetase

The procedure was adapted from an existing protocol^18^. To a reaction mixture in a 1.5 mL microcentrifuge tube containing 10 mM substrate, 10 mM ATP, 20 mM MgCl_2_, 40 mM NH_4_Cl, 25 mM 2-mercaptoethanol in 100 mM imidazole–HCl buffer pH 7.0, recombinant human glutamine synthetase (final concentration: 5 ng/mL) was added. The tubes were incubated in a preheated thermomixer at 37 °C and shaken at 600 rpm (30 s on, 30 s off). 5 μL aliquots were collected in triplicate every 5 min and immediately diluted with 195 μL of cold 60% ACN containing 10 μM Glu-d5 and Gln-d5 as internal standards in a 96 well plate. After collecting all samples, 200 μL of ACN was added to all wells and the plate was shaken at 1000 rpm for 5 min at room temperature, and then stored at − 80 °C until analysis.

### Catalysis of 2MeGln hydrolysis by GLS1 glutaminase

The procedure was adapted from an existing protocol^19^. To a reaction mixture in a 1.5 mL microcentrifuge tube containing 10 mM rac-2MeGln or l-Gln-^13^C2 in 100 mM phosphate pH 7.4 buffer made in 20% atom ^18^O water, recombinant GLS1 (final concentration: 5 ng/mL) was added. The tubes were incubated in a preheated thermomixer at 37 °C and periodically shaken at 600 rpm (30 s on, 30 s off). 5 μL aliquots were collected in triplicate every 5 min and immediately diluted with 195 μL of cold 60% ACN containing 10 μM Glu-d5 and Gln-d5 as internal standards in a 96 well plate. After collecting all samples, the plate was shaken at 1000 rpm for 5 min at room temperature, and then stored at − 80 °C. Before analysis the plate was thawed and 10 μL aliquots of the extracted reaction mixtures were diluted with 190 μL of 90% acetonitrile.

The quantification of the newly formed 2MeGlu in the presence of the preexisting trace amounts was performed in the following way. Peak area ratio of Glu-^13^C2 to Glu-^13^C2^18^O was calculated in all samples containing Glu-^13^C2. The mean ratio value was then applied to calculate the hypothetical peak area of the newly formed 2MeGlu based on the directly measured 2MeGlu-^18^O peak area. The ratio of the newly formed 2MeGlu peak area and the total 2MeGlu peak area was applied to calculate the concentration of the newly formed 2MeGlu from the total 2MeGlu concentration determined directly using the calibration curve.

### Astrocyte transport and metabolism of 2MeGlu

Primary astrocytes from C57Bl/6 mouse brain were cultured in complete astrocyte growth medium at 37 °C at 5% CO_2_ on 96 well plates. Cells were exposed to 1 mM compound in 100 μL medium for 2 h at 37 °C. Supernatants were collected, centrifuged at 2500*g* for 10 min at 4 °C. 10 μL aliquots were diluted with 190 μL 60% ACN containing 10 μM Glu-d5 and Gln-d5. Cells were quickly washed with 250 μL cold PBS twice and suspended in 200 μL of 60% ACN containing 10 μM Glu-d5 and Gln-d5. All samples were shaken at 1000 rpm for 5 min and then stored at – 80 °C.

### Neuronal transport and metabolism of 2MeGln

The procedure was adapted from an existing protocol^67^. Wild-type (WT) C57BL/6J mating pairs were housed according to Stanford IACUC protocol. On day P0, pups were euthanized and brains dissected in cold HBSS (without Ca^2+^ or Mg^2+^). The forebrain was microdissected to isolate the cortex and hippocampus. These brain regions were minced in cold HBSS before trypsinization at 37 °C for 9 min, followed by trituration, through 2 glass polished Pasteur pipettes. The resulting cell suspension was passed through a 100 μm filter to isolate single cells which were sedimented at 250*g* for 5 min. The cell pellet was then resuspended in complete Neural Basal Media (Neural Basal Media + Glutamax + B-27 Plus Supplement + Pen/Strep) + 10%FBS, counted using a hemocytometer and plated at 1 × 10^6^ cells per mL into a 24 well plate (1 mL per well) coated with poly-d-lysine and laminin. Cultures were grown at 37 °C/5% CO_2_ in a tissue culture incubator. Media was exchanged 50% on day 1 in vitro (1DIV) with fresh complete Neural Basal Media without FBS and thereafter every other day with the same media. On 3DIV the media was spiked with 2 μM AraC to limit glial growth. On 10DIV the media was completely replaced with 500 μL Neural Basal Media containing B-27 Plus Supplement + Pen/Strep without added Glutamax but instead containing 1 mM substrate at 37 °C for 2 h. The supernatants were collected, centrifuged at 16,000*g* for 5 min at 4 °C. 10 μL aliquots were diluted with 190 μL 60% ACN containing 5 μM Glu-d5 and Gln-d5. The cells were washed with 500 μL PBS twice and suspended in 200 μL of 60% ACN containing 5 μM Glu-d5 and Gln-d5. All samples were shaken at 800 rpm for 5 min and then stored at −80 °C until analysis.

### In vivo animal studies

Behavioral tests were performed on 2 month-old, male, C57Bl/6 mice. We selected male mice because SBFNL historical controls are male mice. First, we assessed safety and tolerability of acute exposure to our novel chemicals through the following sequence of tests immediately following IP injection: maximum tolerated dose with novel cage observation at 100, 300, or 900 mg/kg, and then SHIRPA, activity chamber, and then hot plate latency using 100 or 30 mg/kg IP in different drug naïve mice. Second, we investigated the impact of chronic exposure to 10 mg/kg (3-times lower dose than that with minimal acute impact on locomotor activity) daily IP injection starting one week prior to assessment of locomotion in the activity chamber and elevated plus maze (week 1), or learning and memory with Y maze (week 2), Morris water maze (weeks 2 and 3), and then fear conditioning (week 4) according to established protocols^25–30^, including not counterbalancing the escape platform location^68,69^. Animals were dosed daily from 3 to 5 PM after the conclusion of each behavioral testing session. While the time from first dose for each behavioral test is different, our experimental protocol allows for efficient testing of chronic effects of novel compounds. See Supplementary Methods for details.

### Approval for animal experiments

All procedures followed the National Institute of Health guidelines and were approved by the Stanford University’s Administrative Panel on Laboratory Animal Care.

## Supplementary Information

Supplementary Information.

## Data Availability

Data generated during this study are available from the corresponding author on reasonable request.
