# Supplementary material for: Enantiomers of 2-methylglutamate and 2-methylglutamine selectively impact mouse brain metabolism and behavior
Source: Sci Rep. 2021 Apr 14;11:8138. doi: 10.1038/s41598-021-87569-1 (PMC8047011; doi:10.1038/s41598-021-87569-1)
Supplement: Supplementary file 1 — Supplementary Information. [file 41598_2021_87569_MOESM1_ESM.pdf]

Supplementary Information

Enantiomers of 2-methylglutamate and 2-methylglutamine selectively impact mouse brain metabolism and behavior

Adam M. Wawro,[1] Chandresh R. Gajera,[1] Steven A. Baker,[1] Robert K. Leśniak,[2] Curt R. Fischer,[2] Nay L. Saw,[3] Mehrdad Shamloo,[3,4] and Thomas J. Montine[1]*

[1]Department of Pathology, Stanford University, Stanford, USA

[2]ChEM-H, Stanford University, Stanford, USA

[3]Behavioral and Functional Neuroscience Laboratory, Stanford University, Stanford, USA

[4]Department of Neurosurgery, Stanford University, Stanford, USA

Corresponding author: tmontine@stanford.edu

Supplementary Figures

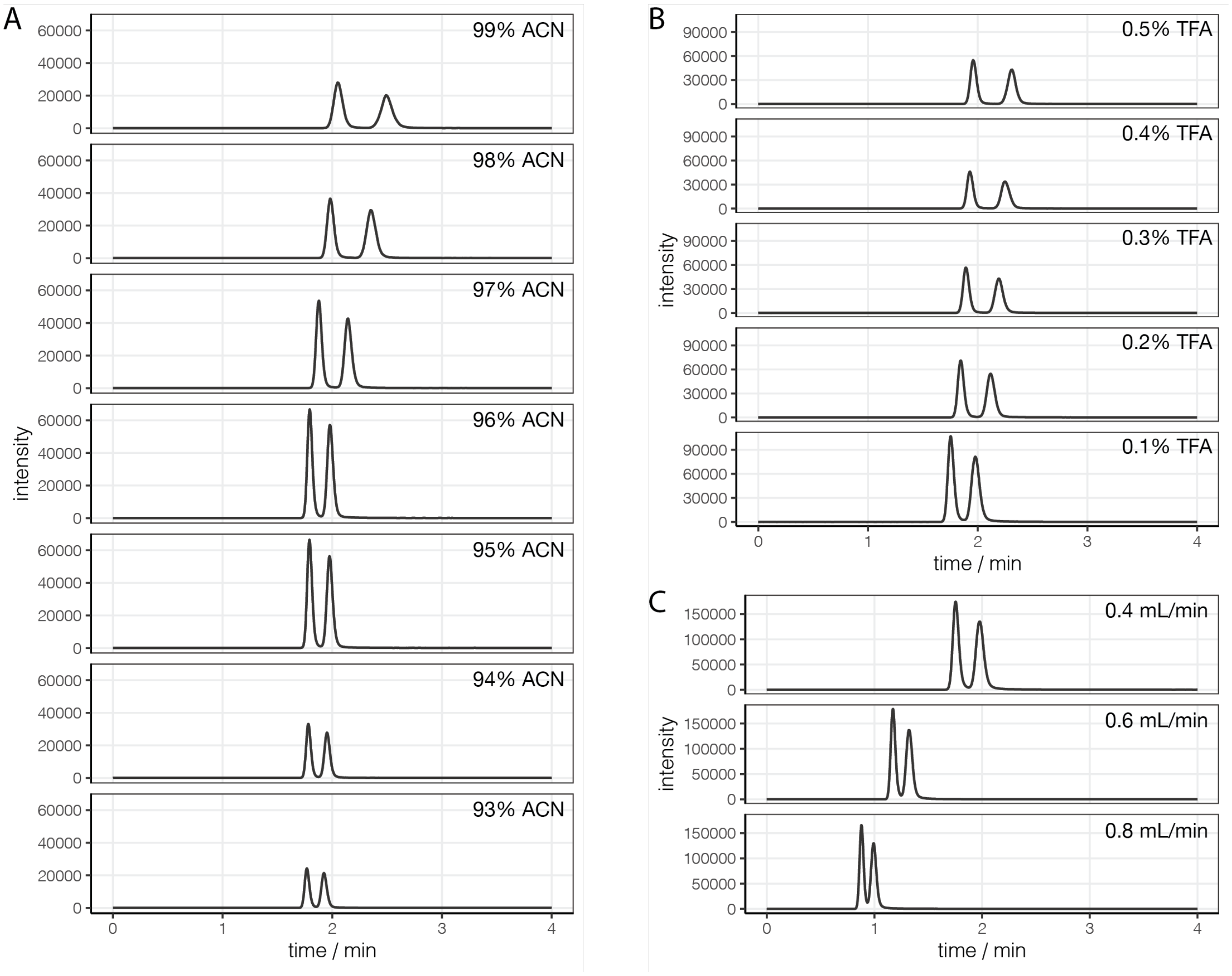

Figure S1. Optimization of 2MeGlu chiral separation. LC-MS/MS system was equipped with a chiral CROWNPAK CR-I(+) column. Acetonitrile (A), trifluoroacetic acid (B) and mobile phase flow (C) were adjusted to achieve the optimum resolution in the minimum time and at the lowest acid concentration. Final conditions: 96% acetonitrile, 0.1% trifluoroacetic acid, flow 0.4 mL/min.

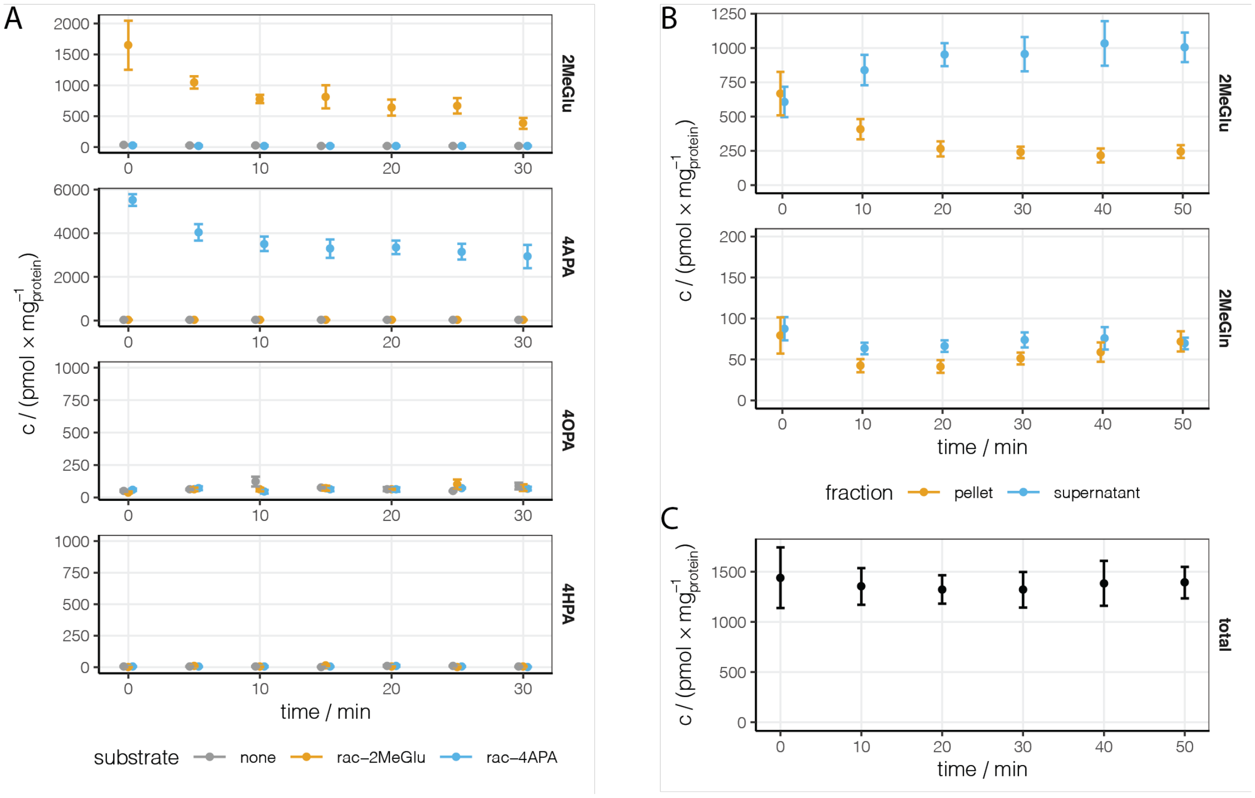

Figure S2. Synaptosomal metabolism of 2MeGlu and 4APA. (A) Synaptosomes were preincubated with 100 µM substrate for 15 min at 37 ºC. The pellet was washed, resuspended in normal KRP buffer and incubated at 37 ºC as the levels of potential metabolites were monitored at 5 min intervals. 4OPA and 4HPA signals were below the limit of quantification. Data are mean ± SEM, n = 3. (B) Synaptosomal metabolism and retention of 2MeGlu. Synaptosomes preincubated with 100 µM 2MeGlu were resuspended in normal KRP buffer and intra- and extrasynaptosomal levels of 2MeGlu and 2MeGln were monitored. (C) Total concentration of intra- and extrasynaptrosomal 2MeGlu and 2MeGln in the monitored samples. Data are shown as mean ± SEM, n = 5.

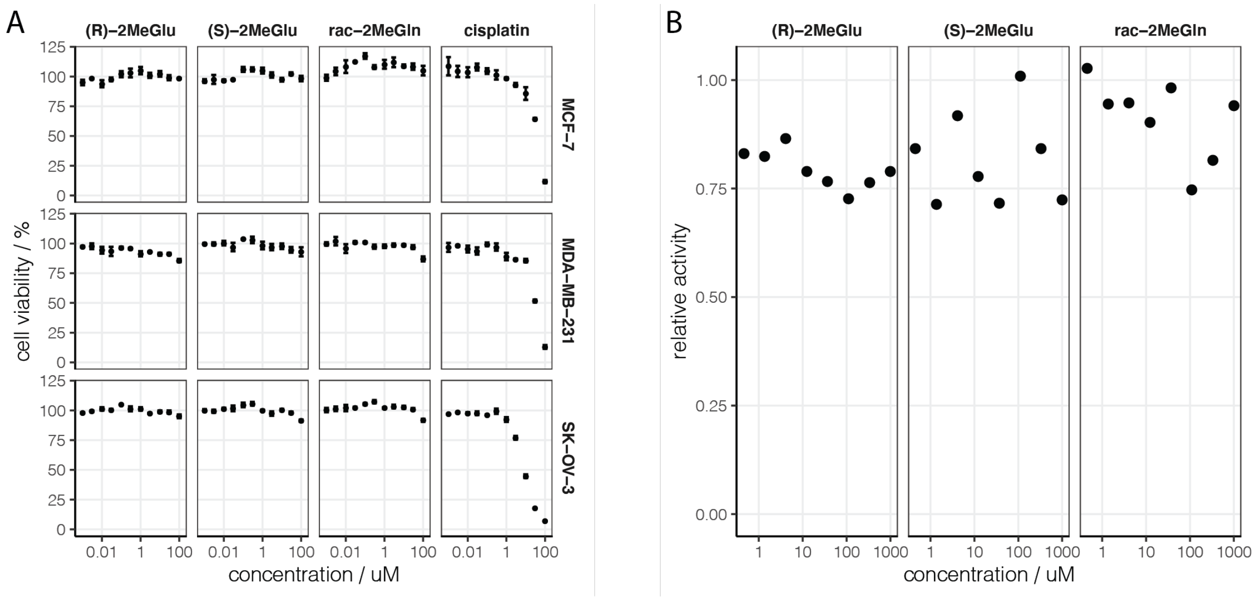

Figure S3. (A) Cell viability assay using 2MeGlu and 2MeGln on MCF-7, MDA-MB-231 and SK-OV-3 cancer cell lines. Compounds were tested at 1 nM – 100 μM range in triplicate with cisplatin as a positive control. (B) GLS1 inhibitor assay using 2MeGlu and 2MeGln in the 0.5 μM – 1 mM range. Data are shown as mean ± SEM, n = 3.

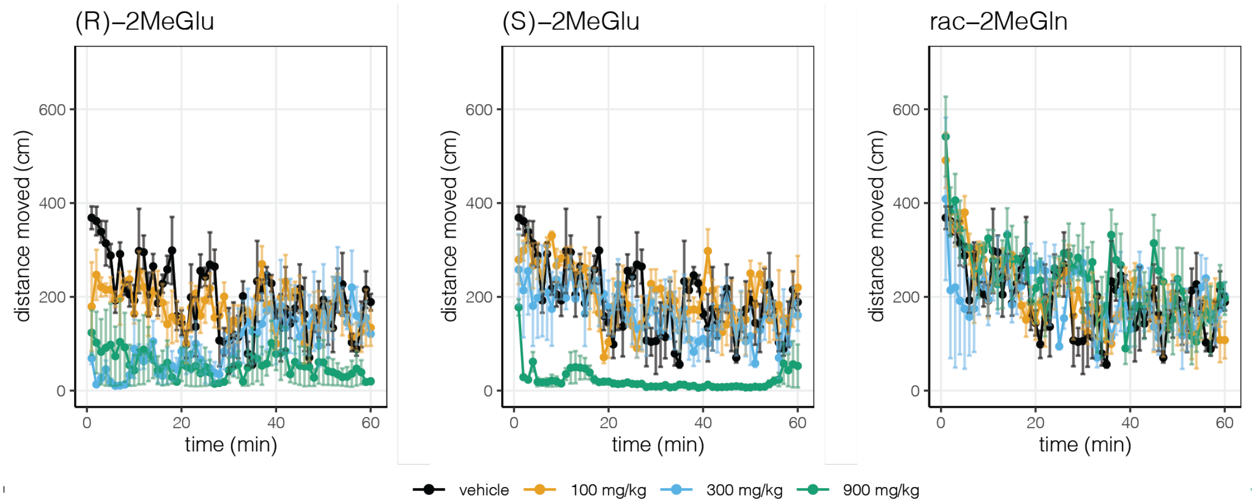

Figure S4. Exploratory and locomotor behavior in 2 month-old male C57Bl/6 mice was assessed by total distance moved in the novel cage test. Data are shown as mean ± SEM (n=3 mice per group) for distance (cm) moved each minute over 1 to 60 minutes following injection. These data were used to create the dose-response graph in Figure 6A.

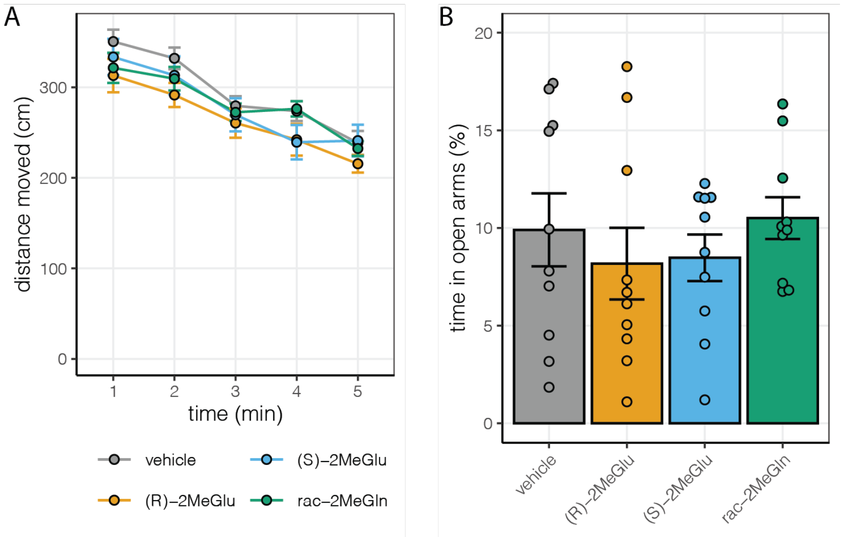

Figure S5. Behavioral effects of chronic 10 mg/kg/day IP dosing in 2 month-old male C57Bl/6 mice. Elevated Plus maze was performed after the first week of chronic exposure. Data are shown as mean ± SEM, n = 10 mice per group. **(A)** Two-way repeated measures ANOVA for distance moved (cm) had $F_{Interaction}$ (12, 144) = 0.9112, P=0.5375; F_{Time} (4, 144) = 66.01, P<0.0001; and $F_{Treatment}$ (3, 36) = 1.076, P=0.3713. **(B)** One-way ANOVA for percentage of time spent in the open arms of the maze vs. treatment measured over 5 minutes had $F_{Treatment}$ = 0.531, P=0.664.

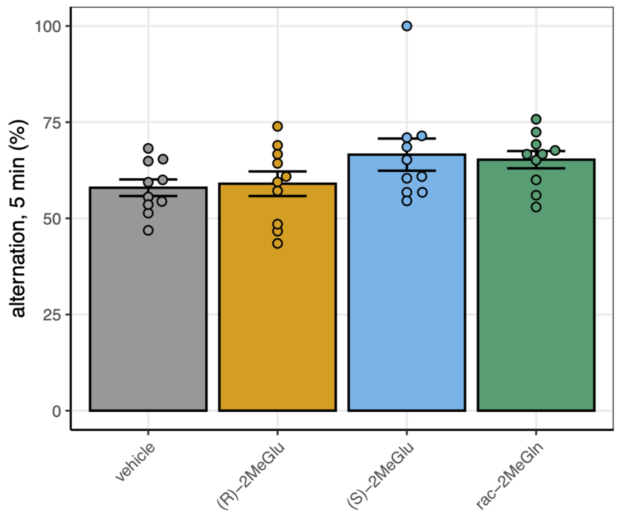

Figure S6. Behavioral effects of chronic 10 mg/kg/day IP dosing in 2 month-old male C57Bl/6 mice. Y maze was performed during the second week of chronic exposure. Percent alternation over 5 minutes is shown as scatter plot as well as mean ± SEM, n = 10 mice per group. One-way ANOVA had $F(3, 36) = 2.018$, $P>0.05$.

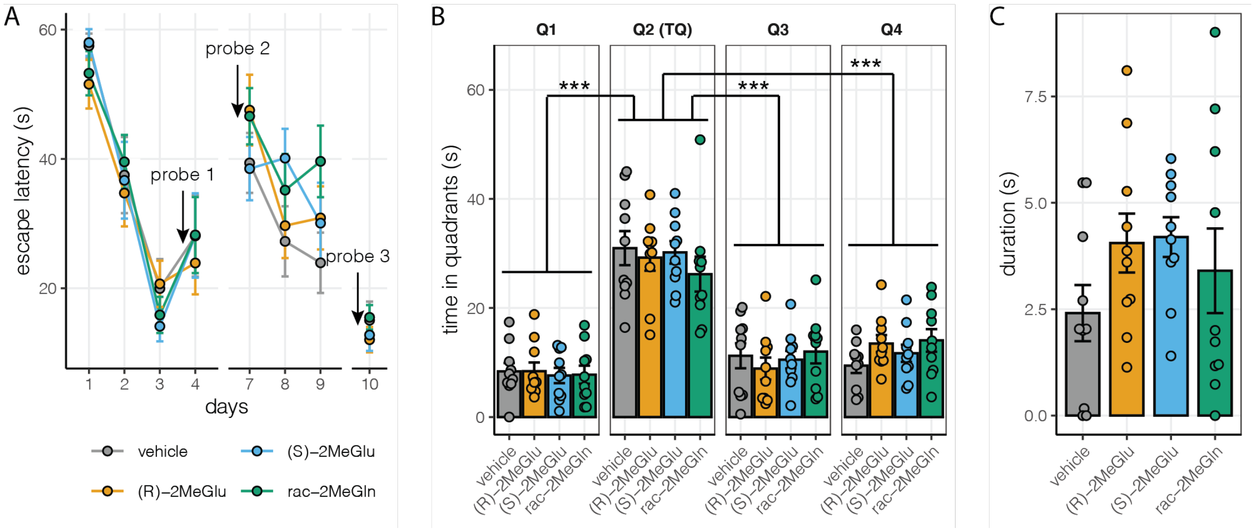

Figure S7. Behavioral effects of chronic 10 mg/kg/day IP dosing in 2 month-old male C57Bl/6 mice. Morris water maze was performed during the second and third weeks of chronic exposure. Data are shown as scatter plot as well as mean ± SEM, n = 10 mice per group. **(A)** Two-way repeated measures ANOVA for escape latency (seconds) had $F_{Interaction}$ (21, 252) = 0.8483, P=0.6582; F_{Time} (7, 252) = 37.39, P<0.0001; and $F_{Treatment}$ (3, 36) = 0.4551, P=0.7153. **(B)** Two-way ANOVA for probe 1 time (seconds) in quadrants had $F_{Interaction}$ (3, 36) = 0.844, P=0.577; $F_{Quadrants}$ (1, 36) = 87.6, ***P<0.0001; and $F_{Treatment}$ (3, 36) = 0.000, P=1.000. **(C)** One-way ANOVA of probe 1 duration in the virtual platform (seconds) had P=0.3066.

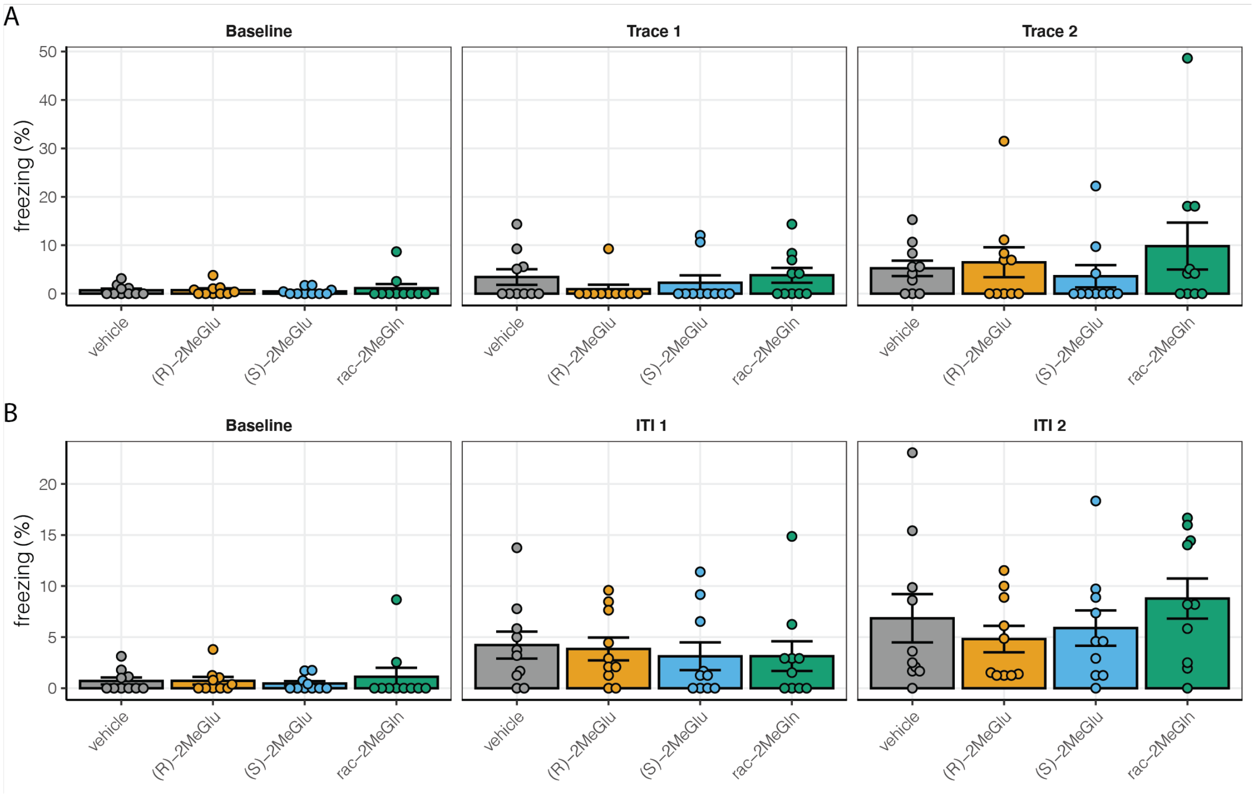

Figure S8. Behavioral effects of chronic 10 mg/kg/day IP dosing in 2 month-old male C57Bl/6 mice in fear conditioning tests on the fourth week of chronic exposure. Fear conditioning paradigm Day 1; training day, all treatment groups showed equivalent learning of the tone-shock association on Day 1. There was no difference between the experimental groups in (A) trace learning or (B) intertrial interval (ITI) freezing during the training day suggesting comparable learning of the task among all experimental groups. All bars represent mean ± SEM, n = 10 mice per group.

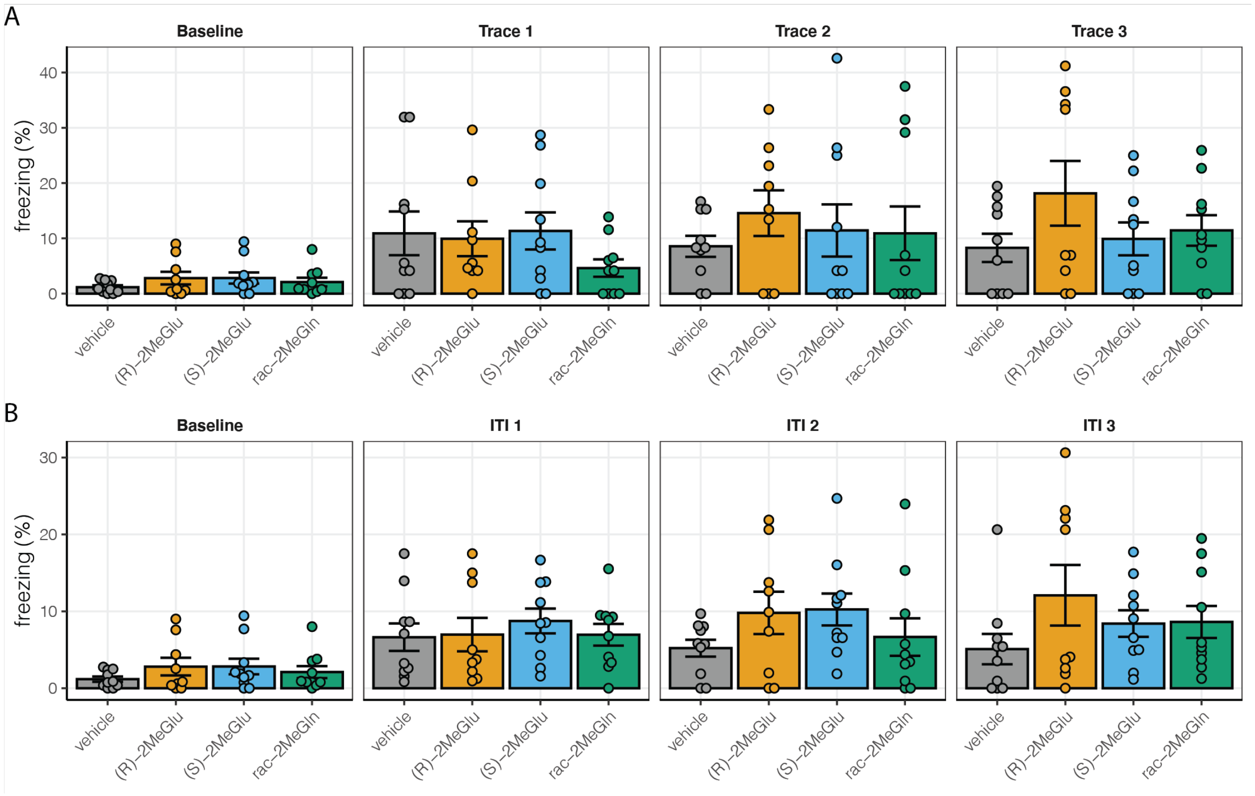

Figure S9. Behavioral effects of chronic 10 mg/kg/day IP dosing in 2 month-old male C57Bl/6 mice in fear conditioning tests on the fourth week of chronic exposure. Fear conditioning paradigm Day 3; Cued recall: there was no significant difference detected in freezing on Day 3 between experimental groups over the three tone presentations as shown in Figure 7B. In addition, no significant difference was observed between the 18 seconds trace periods following 3 tone presentation on Day 3 (Figure S9A). Finally, no significant difference in freezing was detected between experimental groups during the intertrial interval (ITI) on Day 3 following tone presentation, indicating comparable cued recall among experimental groups on day 3 (Figure S9B). All bars represent mean ± SEM, n = 10 mice per group.

Supplementary methods

Materials

rac-2-Methylglutamic acid (cat. no. M0229), and 4-oxopentanoic acid (cat. no. L0042) were obtained from TCI America. rac-4-Hydroxypentanoic acid (as sodium salt, cat. no. EN300-176456) and rac-4-aminopentanoic acid (as hydrochloride, cat. no. EN300-81918), were obtained from Enamine.

LC-MS-grade acetonitrile (cat. no. A955), formic acid (cat. no. A117) and trifluoroacetic acid (cat. no. A116) were obtained from Fisher Scientific. Isotopically labeled L-glutamic acid (2,3,3,4,4-d5, cat. no. DLM-556; 1,2-13C2, cat. no. CLM-2024) and L-glutamine (2,3,3,4,4-d5, cat. no. DLM-1826; 1,2-13C2, cat. no. CLM-2001) were purchased from Cambridge Isotope Laboratories. HPLC-grade ammonium formate (cat. no. 60-020-36) was obtained from Fluka.

C57Bl/6 mouse brain primary astrocytes were purchased from Lonza, Basel, Switzerland (cat. no. M-ASM-330). Astrocytes were cultured in medium (cat. no. CC-3187) and supplemented with growth factors (cat. No CC-4123) to make a complete astrocyte growth medium. The second passage of astrocytes cultured in complete astrocyte growth medium at 37 ^{o}C and 5% CO_2 was used for experiments.

Recombinant human GLS1 (cat. no. SRP0516) was purchased from Sigma. Recombinant human GS (cat. no. ab222354) was purchased from Abcam.

Chemical characterization of key compounds

(R)-2MeGlu: ^{1}H NMR (D_2O, 500 MHz): δ 2.48 (m, 2H), 2.13 (m, 2H), 1.52 (s, 3H). ^{13}C{^{1}H} NMR (D_2O, 126 MHz): δ 177.1, 175.7, 60.7, 31.9, 29.0, 22.1. >99% pure (UPLC–ELSD), 95% e.e. (chiral LC-MS)

(S)-2MeGlu: ^{1}H NMR (D_2O, 500 MHz): δ 2.49 (m, 2H), 2.15 (m, 2H), 1.53 (s, 3H). $^{13}C\{^{1}H\}$ NMR (D_2O, 126 MHz): δ 176.9, 175.5, 60.6, 31.8, 28.9, 22.0. >99% pure (UPLC–ELSD), 95% e.e. (chiral LC-MS)

rac-2MeGln: ^{1}H NMR (D_2O, 500 MHz): δ 2.38 (m, 2H), 2.10 (m, 2H), 1.52 (s, 3H). $^{13}C\{^{1}H\}$ NMR (D_2O, 126 MHz): δ 177.7, 176.0, 60.9, 32.7, 29.9, 22.2. >97% pure (UPLC–ELSD).

Protein concentration determination

500 μL of the synaptosome preparation was centrifuged (8,000g for 4 min at 4 °C) and the supernatant was discarded. The pellet was suspended in 200 μL of RIPA buffer and incubated in ice for 90 min. The suspension was then sedimented (14,000g for 10 min at 4 °C) and the supernatant used to determine the total protein concentration using Pierce BCA Protein Assay Kit and Biotek Epoch microplate spectrophotometer by measuring absorbance at 562 nm.

LC-MS/MS analysis, HILIC conditions

LC-MS/MS HILIC analysis was performed using Agilent 6470 Triple Quadrupole LC/MS System with Agilent 1290 Infinity II LC module. Prior to analysis all samples were filtered through a Multiscreen Solvinert 0.45 μm PTFE filter plate to a polypropylene 0.5 mL 96-well plate. 2.5 μL of the sample was injected onto an Acquity UPLC BEH Amide column (2.1 × 50 mm, 1.7 μm particle size, Waters, part no. 186004800) equipped with an Acquity UPLC In-Line Filter (Waters, part no. 205000343), thermostatted at 40 °C. The sample was eluted at a flow rate of 0.6 mL min^{-1} with one of three gradient methods. Method 1 was developed for rapid quantification of all investigated compounds in relatively diluted biological matrices (synaptosome extract, cell extracts, in vitro reaction supernatants), and used lower-pH buffer A (5 mM ammonium formate in water containing 0.1% formic acid, pH 3.2) and buffer B (5 mM ammonium formate in 95:5 (v/v) acetonitrile–water containing 0.1% formic acid) with the following gradient: 0.0–2.5 min: linear gradient from 10% to 27.5% A; 2.51–3.0 min: hold 45% A;

3.01–3.5 min: hold at 10% A. Method 2 was a modified version of Method 1 for accurate quantification of small amounts of 2MeGlu in brain tissue homogenate, where two isomeric analytes were found to partially overlap with the peak of interest and interfere with quantification. It used the same buffer system and a longer gradient method: 0.0–5.0 min: linear gradient from 0% to 32.5% A; 5.01–6.0 min: hold 0% A. The mass spectrometer was operated in multiple reaction monitoring (MRM) positive mode with capillary voltage set to 3.5 kV, nebulizing gas held at 250 °C, and superheated sheat gas held at 300 °C. In experiments where isotopically labeled internal standard was added, results were corrected accordingly.

LC-MS/MS analysis, chiral conditions

LC-MS/MS chiral analysis was performed using Agilent 6470 Triple Quadrupole LC/MS System with Agilent 1290 Infinity II LC module. Prior to analysis all samples were filtered through a Multiscreen Solvinert 0.45 µm PTFE filter plate to a polypropylene 0.5 mL 96-well plate. 1.0 µL of sample was injected onto an CROWNPAK CR-I(+) column (3.0 × 150 mm, 5 µm particle size, Daicel, part no. 53784) equipped with an Acquity UPLC In-Line Filter (Waters, part no. 205000343), thermostatted at 20 °C. The sample was eluted isocratically at a flow rate of 0.4 mL min^{-1} with buffer C (96:4 ACN/water v/v, 0.1% TFA; parameters varied in the optimization experiments). The mass spectrometer was operated in multiple reaction monitoring (MRM) positive mode with capillary voltage set to 3.5 kV, nebulizing gas held at 250 °C, and superheated sheat gas held at 300 °C.

Synaptosome preparation

Cerebral hemispheres from a C57BL/6 mouse were homogenized in 3 mL of Buffer S using pre-chilled 5 mL Potter-ELV tissue grinder (800 rpm, 8 strokes). The crude homogenate was diluted with Buffer S to the total volume of 4 mL and centrifuged at 1,000g for 10 min at 4 °C. The supernatant was collected and sedimented (10,000g for 20 min at 4 °C). The supernatant was discarded and the pellet washed by resuspension–centrifugation (8,000g for 4 min at 4 °C) with

Buffer S (4 mL, × 1) and Buffer KRP (2 mL, × 3). The resulting pellet was resuspended in 3 mL of Buffer KRP and filtered on Ultrafree-CL 5 µm PVDF centrifugal device (1,000g for 4 min at 4 °C). The filtrate was diluted to 15 mL with ice cold Buffer KRP and kept on ice. The synaptosome preparation was used immediately in the following experiments.

Synaptosomal neurotransmitter uptake assay

96-well plate experiments: 250 µL aliquots of the synaptosome suspension were transferred to two 0.8-mL 96 well plates, one on ice (0 ºC samples only) and one in a thermoblock (37 ºC samples only), and equilibrated for 10 min. 5 µL of a test compound (0.5 mM; final concentration: 10 µM) in Buffer KRP or Buffer KRP blank were added 10, 20 or 30 min before the incubation termination. "0 min" samples were prepared by placing the test compound solution in a separate pre-chilled V-shape bottom 2-mL 96 well plate; at the end of incubation 200 µL aliquots of all samples were transferred to the 2-mL 96 well plate and centrifuged (2,500g for 15 min at 4 °C) immediately. The resulting pellets were rinsed with Buffer KRP, followed by centrifugation, twice, then resuspended in 200 µL of 60% ACN containing 2.5 µM Glu-d5 as internal standard and stored at –80 °C until analysis.

Pharmacokinetics on 2MeGlu and 2MeGln in mice

C57BL/6 mice (Jackson Laboratory cat. no. 000664) were administered 100 mg/kg IP. At the times indicated, mice were deeply anesthetized by isoflurane, blood was collected by cardiac puncture and left to clot on ice, and then animals were perfused with cold PBS until viscera turned pale and blood ceased flowing from the inferior vena cava. Whole brain was promptly dissected and one half frozen at –80 ºC until homogenization in ice cold PBS (pH 7.4) using a probe ultrasonic homogenizer (Branson Sonifier 450, Power: 5, Cycle: 30%, time: 10 s) on ice.

The lysate was centrifuged (2,500g for 15 min at 4 °C) and the supernatant was assayed using Pierce BCA Protein Assay Kit (23225) using BioTek Epoch plate spectrophotometer by recording absorbance at 562 nm.

Two or three 10 µL aliquots of the homogenate were transferred to a 96 well plate and extracted with 190 µL of 60% acetonitrile in water containing 10 µM GABA-d6 or 10 µM Glu-d5 and Gln-d5 as internal standards. 40 µL aliquots of the extracts were then diluted with 120 µL of acetonitrile and prepared for LC-MS/MS analysis. Brain and serum half-lives were calculated using the 'PKNCA' package for R. Endogenous levels of Glu, Gln and GABA were determined in the brains of mice used in the pharmacokinetics experiment. MRM transitions specific for Glu, Gln and GABA were monitored simultaneously.

<u>In vivo animal studies; Maximum Tolerated Dose (MTD) and behavioral studies</u>

Animals: Two month-old, naïve male C57Bl/6 mice from Jackson Laboratory (Stock ID# 000664) were group-housed in a Stanford University animal facility with 12:12 hour light/dark cycle (8:30 am light off 8:30 am light on) and had free access to water and food. All behavioral tests were conducted during the animal dark-cycle. All procedures followed the National Institute of Health guidelines and were approved by the Institutional Administrative Panel on Laboratory Animal Care (APLAC).

Dosing: (R)-2MeGlu (30, 100, 300, or 900 mg/kg), (S)-2MeGlu (30, 100, 300, or 900 mg/kg), rac-2MeGln (30, 100, 300, or 900 mg/kg) and vehicle (PBS) were administered at 10ml/kg IP dosing volume. Three mice were dosed once with the assigned drug (100, 300, or 900 mg/kg) or vehicle and immediately placed in a new cage for novel cage observation. Subsequently, six mice were dosed once with the assigned drug (30 or 100 mg/kg) or vehicle and tested by SHIRPA, activity chamber, and then hot plate tests.

Novel Cage Observation: The mouse was dosed and immediately placed in the center of a clean, Innovive disposable rat cage with bedding (dimensions: 17"L x 13.4"W x 7.8"H) and allowed to move freely while being tracked automatically. Mice were monitored with Ethovision XT tracking software for the distance moved and average velocity during the 60-minute trial. They were returned to their home cage after the test. A new cage was used for each mouse.

Hot plate test: Hot Plate apparatus (IITC Inc. Model 39) was set to 55°C± 0.2. On testing day, mice were placed on the surface of the hot plate and covered by a transparent glass cylinder (25cm high and 12cm diameter). A 30 second cutoff time was assigned. A remote foot-switch pad was used to control the start/stop/reset function. Latency time was recorded when hind paw licking or jumping off first occurred.

SHIRPA: The SHIRPA preliminary screen designed by Rogers *et al.* (1997) was modified and used to assess the general health and behavioral traits of the mice for the initial five minutes after drug administration. The test was conducted in three different testing locations: A cylindrical clear plastic viewing jar (15 cm height x 10 cm diameter), a polycarbonate cage (Arena) with 10 cm x 10 cm squares on the floor, and a 1 cm x 1 cm wire grid. The test began by placing the mouse in the clear plastic viewing jar for 60 seconds, and the behavioral parameters were monitored and recorded. The mouse was then transferred to an open-top arena by releasing them from 25 cm above the floor of the arena. Upon completion of monitoring the parameters inside the arena, the mouse was removed from the arena by the tail. Behavioral parameters monitored were according to Portmann *et al.* (2014).

Activity Chamber Open Field: Assessment was made in an Open Field Activity Arena (Med Associates Inc., St. Albans, VT. Model ENV-515) mounted with three planes of infrared detectors within a specially designed sound-attenuating chamber (Med Associates Inc., St. Albans, VT. MED-017M-027). The arena was 43 cm (L) x 43 cm (W) x 30 cm (H) and the

sound-attenuating chamber was 74 cm (L) x 60 cm (W) x 60 cm (H). The mice were placed in the corner of the testing arena and allowed to explore the arena for 60 min while being tracked automatically. Parameters measured include distance moved, velocity, rearing, and times spent in the periphery and center of the Arena. Periphery was defined as the zone 5 cm away from the arena wall. The Arena was cleaned with a 1% Virkon solution at the end of the trial.

The novel cage observation (*vide supra*) and activity chamber open field assessment are not performed in a similar manner in our laboratory, and do not represent the same behavioral outcome. The novel cage protocol is conducted on the benchtop in a lighted room using a clean home cage, and tests habituation using video recording and scoring of behavioral performance. Moreover, the novel cage allows the tester to visualize any other side effects such as seizure or repetitive jumping. The activity chamber is in an enclosed box, and the test is done in the dark, without bedding, and in a sound isolating chamber. The activity chamber measures distance moved using infrared beams and without video recordings.

In vivo animal studies; Chronic Efficacy Study using C57Bl/6J mice

Animal: Two month-old, naïve male C57Bl/6 mice from Jackson Laboratory (Stock ID# 000664) were used in the study. The animals were housed as described above.

Dosing: (R)-2MeGlu (10 mg/kg), (S)-2MeGlu (10 mg/kg), rac-2MeGln (10 mg/kg), and vehicle (PBS) were administered IP at 10 ml/kg dosing volume daily. The first dose was administered one week prior to behavioral tests and continued for four weeks during the behavioral testing.

Activity Chamber Open Field: The assessment took place in an Open Field Activity Arena as described above for 30 min. The Arena was cleaned with a 1% Virkon solution at the end of the trial. Activity chamber open field assessment was conducted after one week of exposure to compounds or vehicle.

Elevated Plus Maze: The maze was made of acrylonitrile butadiene styrene (ABS) plastic and had two open arms and two close arms that were 30 cm long and 5 cm wide. The center area where the open and close arms meet was 5 cm x 5 cm. The open arms had 2 mm lips at the edges, and the closed arms had 15 cm opaque walls. The maze was elevated 50 cm and surrounded by privacy blinds during the test. The maze was illuminated to 7 lux using red light throughout the test. Each mouse was released in the center of the maze and given 5 min to explore the maze. Duration and frequency in each zone of the maze were recorded using Ethovision (Noldus Information Technology, Wageningen, the Netherlands) tracking software. The maze was cleaned with a 1% Virkon solution at the end of the trial. Elevated plus maze was conducted after one week of exposure to compounds or vehicle.

Y Maze Spontaneous Alternation: The Y maze is made of plastic with 3 arms in a "Y" shape. The arms are labelled as Arm A, B, and C; Arm A is 20.32 cm (L) x 5 cm (W) 12.7 cm (H), Arm B and C are 15.24 cm (L) x 5 cm (W), and 12.7 cm (H). The test is based on the willingness of rodents to explore a novel environment and designed to measure spontaneous alternations in rodents. Each mouse was placed in the center of the maze facing Arm B. The first entry was excluded from data analysis because the animals were led to the initial arm entry by the experimenter. Using an overhead camera, the number of arm entries and alternations were recorded for 5 minutes. An arm entry is when all four paws enter into a new arm of the maze. The apparatus was cleaned with 1% Virkon. Parameters recorded were % alternation and the total number of entries into arms. The Y maze test was conducted after two weeks of exposure to compounds or vehicle.

Morris Water Maze: During the Hidden Platform Training (HPT), mice were given 2 trials, with 1-minute inter-trial-intervals (ITIs) in a circular water tank (172 cm diameter) filled with opaque water at 22.0 ± 1.5°C. Nontoxic tempera paint was used to make the water opaque. A circular platform (17 cm diameter) was submerged 1 cm below the water's surface and placed in one of

the four quadrants of the pool (Quadrant 2). The release locations into the pool were randomly chosen to prevent spatial bias and the platform was also relocated during reversal training. We conducted 3 days of training with 2 trials each day for a total of the 6 trials prior to probe 1 (Days 1 to 3). Specifically, mice were given a maximum of 60 s to find the submerged platform per trial. The experimenter guided the mice to the platform if they failed to find it within the 60 s. After remaining on the platform for 15 s, mice were removed from the platform and placed in a dry cage with a paper towel. This process was repeated for 3 days. On the following day (~24 hours after the last training trial), the platform was removed from the pool, and a 60 s probe trial was conducted. After acquiring the data for the probe trial, the platform was replaced at the same location, and the mice were given additional two trials of training. Three days (~72 hours) after the last training trial, a second probe trial was conducted. The platform was relocated to a new quadrant (Quadrant 4) after the second probe trial, and the animals were trained in Reversal Hidden Platform Training (RHPT). The procedures for RHPT were identical to HPT and were conducted for 3 days (Days 7 to 9). A third probe trial was conducted on the following day (~24hr). The platform was relocated to Quadrant 1 upon completion of the third probe trial. A ping pong ball was erected from the submerged platform and the mice were given two trials of Visible Platform Training to ensure they lacked gross sensorimotor deficit and visual impairment. The escape latency, distance moved, duration in zones, and velocity of the animals were recorded using Ethovision XT (Noldus Information Technology, Wageningen, Netherlands). The Morris water maze test was conducted during two to three weeks of exposure to compounds or vehicle.

Fear Conditioning: Coulbourn Instruments (Holliston, MA, USA) fear conditioning system and FreezeFrame software were used for analysis. The experiment consisted of 1 day of training, 1 day of contextual testing, and 1 day of cued testing. The training and contextual testing chambers were identical with similar context. The walls were made of aluminum. The floor was

a gray metal grid through which the US was delivered, and the chambers used yellow color house light and were scented with mint extract to produce a unique smell in the chambers. The chambers were cleaned with a 10% simple green solution (Sunshine Makers, Inc.) between each mouse. The cued testing chambers were circular-shaped, made of plastic, used blue color house light, and were scented with vanilla extract to create a unique context different from training and contextual testing. This chamber was cleaned with 70% ethanol between each mouse. Both chambers were mounted within specially designed sound-attenuating chambers. Each chamber had speakers mounted on the wall and included an exhaust fan and camera. On Day 1 training, mice were placed individually into a training chamber for 200 seconds. A tone (20 seconds, 80 dB, 2 kHz) was presented to the mouse, followed by an electrical shock (intensity 0.5 mA, 2 seconds) 18 seconds after the end of the tone (Misane *et al.* 2005, Burman 2014, Lugo *et al.* 2014). This procedure was repeated two times with a 60 second interval from the end of the shock to the next tone. The mouse was removed from the chamber and returned to the home cage 60 seconds after the last shock. On Day 2, the mice were placed back into the training chamber without any tone or shock for contextual memory testing for 15 minutes. On Day 3, the mice were placed into the cued testing chamber with just tones (20 seconds, 80dB, 2 kHz) presented 3 times with 80 second intervals after 200 seconds of habituation. Overall, the mice received the tone and shock pairings only on the training day. An overhead camera was used to record mice freezing behavior. The fear conditioning test was conducted after four weeks of exposure to compounds or vehicle.

We have shown when studying the cognitive enhancement in normal, young control mice, overtraining of the animals will lead to saturation of behavioral learning, making it very difficult to display pharmacological improvements in behavioral paradigms. We therefore chose less training trials per day and less number of training days in the Morris water maze to increase the likelihood of observing drug effects. Likewise, the fear conditioning task, we chose lower shock

intensity and lower number of tone and shock (2x) pairings to create a greater opportunity to observe pharmacologic activity.

Receptor functional & binding assays

Functional assays of all $GABA_A$, NMDA (GluN2A–GluN2D) and AMPA (GluA2) receptor subunits were performed by SB Drug Discovery as a part of their CNS discovery panel using FLIPR technology. Briefly, receptor-expressing HEK 293 cell lines were incubated in a red membrane potential dye in HEPES:HBSS pH 7.4 buffer and the fluorescence (λ_{ex}: 488, λ_{ex}: 510–570) was monitored over time as the test compounds and/or reference compounds were added to the solution. In agonist assay, only test compounds were added. In positive allosteric modulator assay, cells were preincubated with test compounds and then low concentration (approximately EC_{20}) of an appropriate agonist was added. In antagonist assay, cells were preincubated with test compounds and then high concentration (approximately EC_{80}) of an appropriate agonist was added. For $GABA_A$ assays, GABA was used as the agonist, picrotoxin was used as the reference antagonist and allopregnanolone was used as the reference positive allosteric modulator (PAM). For NMDA assays, a mixture of glutamate, glycine and $CaCl_2$ was used as the agonist, MK801 was the reference antagonist and pregnenolone sulfate or GNE 9278 were used as reference PAMs. For AMPA assays, glutamate was used as the agonist, CNQX was the reference antagonist and cyclothiazide was used as the reference PAM. All compounds were tested at 7 concentration points in triplicate and EC50/IC50 was determined where applicable.

Functional $GABA_B$ assay was performed by Eurofins Discovery (Cerep) using Ca^{2+}-dependent fluorimetry in RBL cells expressing human $GABA_B$ (B1/B2) receptor. All compounds were tested at a single concentration of 100 uM in triplicate.

Nonspecific AMPA, kainate and NMDA binding assays were performed by Eurofins Discovery (Cerep) using rat cerebral cortex membranes and radiolabeled [^{3}H]AMPA, [^{3}H]kainic acid and [^{3}H]CGP 39653, respectively. All compounds were tested at a single concentration of 100 uM in triplicate.

mGluR1, mGluR2, mGluR5 and $GABA_B$ binding assays were performed by Eurofins Discovery. mGluR1 assay was performed using rat cerebellum membranes and radiolabeled [^{3}H]quisqalic acid. mGluR2 assay was performed using recombinant Chem-1 cells and [^{3}H]LY341495. mGluR5 and $GABA_B$ binding assays were performed using recombinant CHO-K1 cells and [^{3}H]CGP-54626 and [^{3}H]quisqalic acid, respectively. All compounds were tested at a single concentration of 100 uM in triplicate.

<u>Cancer cells proliferation assay</u>

Cancer cell proliferation assays on MDA-MB-231, SK-OV-3 and MCF-7 cell lines were performed by BPS Bioscience Inc., San Diego, CA. Potential inhibitors were tested in triplicate in 1 nM – 100 μM concentration range with cisplatin as control compound. The incubation time was set to 72 h and the cell proliferation was assayed with the CellTiter-Glo assay from Promega.

<u>GLS1 inhibition assay</u>

GLS1 inhibition assays were performed using GLS1 Inhibitor Screening Assay Kit from BioVision Inc, Milpitas, CA. Potential inhibitors were tested in 0.5 μM – 1 mM concentration range using initial reaction rate measurement.

Supplementary Tables

Table S1: EC50 in mM determined in functional agonist receptor assays. Top test compound concentration was 100 µM (glutamate receptors) or 1 mM (GABA receptors). Assays were performed by SB Drug Discovery using FLIPR technology in stably receptor expressing cells.

receptor	species	(R)-2MeGlu	(S)-2MeGlu
GluN2A	human	ND	ND
GluN2B	human	ND	ND
GluN2C	human	ND	ND
GluN2D	human	ND	ND
GluA2	human	ND	ND
$GABA_A$ α1β1γ2	human	ND	ND
$GABA_A$ α1β2γ2	human	ND	ND
$GABA_A$ α1β3γ2	human	ND	ND
$GABA_A$ α2β1γ2	human	ND	ND
$GABA_A$ α2β2γ2	human	0.66	0.53
$GABA_A$ α2β3γ2	human	0.622	0.342
$GABA_A$ α3β1γ2	human	0.742	0.782
$GABA_A$ α3β2γ2	human	1.043	0.961
$GABA_A$ α3β3γ2	human	0.959	0.725
$GABA_A$ α4β1γ2	human	0.87	0.704
$GABA_A$ α4β2γ2	human	1.246	1.025
$GABA_A$ α4β3γ2	human	>1.000	>1.000
$GABA_A$ α4β3δ	human	ND	ND
$GABA_A$ α5β1γ2	human	ND	ND
$GABA_A$ α5β2γ2	human	ND	ND
$GABA_A$ α5β3γ2	human	ND	ND

$GABA_A$ α6β1γ2	human	0.772	0.28
$GABA_A$ α6β2γ2	human	ND	ND
$GABA_A$ α6β3γ2	human	>1.000	0.998

Table S2: EC50 in mM determined in functional positive allosteric modulator receptor assays. Top test compound concentration was 100 µM (glutamate receptors) or 1 mM (GABA receptors). Assays were performed by SB Drug Discovery using FLIPR technology in stably receptor expressing cells.

receptor	species	(R)-2MeGlu	(S)-2MeGlu
GluN2A	human	ND	ND
GluN2B	human	ND	ND
GluN2C	human	ND	ND
GluN2D	human	ND	ND
GluA2	human	ND	ND
$GABA_A$ α1β1γ2	human	ND	ND
$GABA_A$ α1β2γ2	human	ND	ND
$GABA_A$ α1β3γ2	human	ND	ND
$GABA_A$ α2β1γ2	human	ND	ND
$GABA_A$ α2β2γ2	human	ND	ND
$GABA_A$ α2β3γ2	human	ND	ND
$GABA_A$ α3β1γ2	human	ND	ND
$GABA_A$ α3β2γ2	human	ND	ND
$GABA_A$ α3β3γ2	human	ND	ND
$GABA_A$ α4β1γ2	human	ND	ND
$GABA_A$ α4β2γ2	human	ND	ND
$GABA_A$ α4β3γ2	human	ND	ND
$GABA_A$ α4β3δ	human	ND	ND
$GABA_A$ α5β1γ2	human	ND	ND
$GABA_A$ α5β2γ2	human	ND	ND
$GABA_A$ α5β3γ2	human	ND	ND
$GABA_A$ α6β1γ2	human	ND	ND

$GABA_A$ α6β2γ2	human	ND	ND
$GABA_A$ α6β3γ2	human	ND	ND

Table S3: IC50 in mM determined in functional antagonist receptor assays. Top test compound concentration was 100 µM (glutamate receptors) or 1 mM (GABA receptors). Assays were performed by SB Drug Discovery using FLIPR technology in stably receptor expressing cells.

receptor	species	(R)-2MeGlu	(S)-2MeGlu
GluN2A	human	0.069	ND
GluN2B	human	ND	ND
GluN2C	human	ND	ND
GluN2D	human	ND	ND
GluA2	human	ND	ND
$GABA_A$ α1β1γ2	human	ND	ND
$GABA_A$ α1β2γ2	human	ND	ND
$GABA_A$ α1β3γ2	human	ND	ND
$GABA_A$ α2β1γ2	human	ND	ND
$GABA_A$ α2β2γ2	human	ND	ND
$GABA_A$ α2β3γ2	human	ND	ND
$GABA_A$ α3β1γ2	human	ND	ND
$GABA_A$ α3β2γ2	human	ND	ND
$GABA_A$ α3β3γ2	human	ND	0.978
$GABA_A$ α4β1γ2	human	ND	ND
$GABA_A$ α4β2γ2	human	ND	ND
$GABA_A$ α4β3γ2	human	ND	ND
$GABA_A$ α4β3δ	human	ND	ND
$GABA_A$ α5β1γ2	human	ND	ND
$GABA_A$ α5β2γ2	human	ND	ND
$GABA_A$ α5β3γ2	human	ND	ND
$GABA_A$ α6β1γ2	human	ND	ND

$GABA_A$ α6β2γ2	human	ND	ND
$GABA_A$ α6β3γ2	human	ND	ND

Table S4: Summary of binding inhibition assays. All compounds were tested at 100 μM in triplicate. Values indicate % of radioligand binding inhibition by test compounds. Assays were performed by Eurofins Discovery using radiolabeled ligands and human receptor expressing cells or rat brain membrane preparations.

**receptor**	**species**	**(R)-2MeGlu**	**(S)-2MeGlu**
$GABA_B$ nonsel	rat	5	5
$GABA_B$ (B1a/B2)	human	18	6
$GABA_B$ (B1b/B2)	human	13	14
mGluR1	rat	-3	6
mGluR2	human	-3	25
mGluR5	human	-20	11
mGluR5	human	-2	-4
NMDA nonspec	rat	8	3
AMPA nonspec	rat	15	12
kainate nonspec	rat	18	20

Table S5: Summary of functional assays. All compounds were tested at 100 μM in triplicate. Values indicate % of agonist response. Assays were performed by Eurofins Discovery using Ca^{2+}-dependent fluorimetry and human receptor expressing cells.

**receptor**	**species**	**(R)-2MeGlu**	**(S)-2MeGlu**
$GABA_B$ (B1/B2)	human	0	0
